# Advances in mercury(II)-salt-mediated cyclization reactions of unsaturated bonds

**DOI:** 10.3762/bjoc.17.153

**Published:** 2021-09-09

**Authors:** Sumana Mandal, Raju D Chaudhari, Goutam Biswas

**Affiliations:** 1 Department of Chemistry, Cooch Behar Panchanan Barma University, Panchanan Nagar, Vivekananda Street, Cooch Behar – 736101, West Bengal, India

**Keywords:** carbocyclic, catalyzed, cyclization, heterocyclic, mercury(II) salts

## Abstract

The synthesis of complex cyclic compounds is extremely challenging for organic chemists. Many transition-metal-salt-mediated cyclizations are reported in literature. Hg(II) salts have been successfully employed in cyclizations to form complex heterocyclic and carbocyclic structures that are impossible to synthesize with other transition metal salts. In this review, we have summarized cyclization reactions that are performed with Hg(II) salts. These salts are also successfully applied in stoichiometric or catalytic amounts to form complex cyclic structures and natural products.

## Introduction

The use of transition metal reagents has found considerable applications in organic synthesis [1–4] and has radically changed the realm of chemical science. It also provides a powerful tool for the construction of complex molecular frameworks [5–7]. A plethora of reviews involving transition metals such as Pd(II) [8–10], Ru(II) [11–13], Rh(III) [14–16], Mn(II) [17–19], Au(II/I) [20–22], Ag(I) [23–25] etc. in both cascade and sequential reactions have been published. Electrophilic Hg(II) salts are important reagents in organic synthesis and there is published literature establishing this fact [26–29]. However, the main drawback of Hg(II) salts, as compared to other transition metal salts, is their increased toxicity [30–31]. Hg(II) salts on the other hand, are very cheap in comparison to other transition metal salts (Table 1) and one of the soft Lewis acids of the periodic table [32]. Hg(II) salts have already manifested some unique reactivity and therefore attracted increasing interest from chemists [33]. Many examples involving Hg(II) salts with unsaturated bonds in presence of various nucleophiles giving rise to various types of products are abound in the literature. Utilization of Hg(II) salts in the intramolecular cationic cyclization of olefinic, acetylenic, and allenic substrates having aromatic rings, nucleophiles, and heteroatoms in the neighborhood were well documented. Hg(II) reagents were also often employed in the important cyclization step during total syntheses of many natural products [34]. Despite its wide application in ring formation reactions, only a few review articles on Hg(II)-salt-mediated cyclization reactions are available in the literature [35]. This review describes the intramolecular cyclization of unsaturated compounds in the presence of stoichiometric/catalytic amounts of Hg(II) salts. The classification of this review is based on the following topics.

- Cyclization reactions involving the stoichiometric amount of Hg(II) salts.
- Cyclization reactions involving the catalytic amount of Hg(II) salts.
- Total synthesis involving Hg(II)-salt-mediated cyclization reactions.

**Table 1:** Comparison of price of reagent grade Hg(II) salts with other transition metal salts.

Name of salts	CAS No.	Price $/g^a^

mercury(II) chloride HgCl_2_	7487-94-7	0.67^b^
mercury(II) acetate Hg(OAc)_2_	1600-27-7	1.35^b^
mercuric triflate Hg(OTf)_2_	49540-00-3	12.7^c^
mercury(II) perchlorate Hg(ClO_4_)_2_	304656-34-6	2.83^c^
auric chloride AuCl_3_	13453-07-1	112^d^
silver perchlorate AgClO_4_	7783-93-9	8.24^c^
platinum chloride PtCl_2_	10025-65-7	115^d^

^a^Prices are from http://www.sigmaaldrich.com (as on 05-08-2021). ^b^100 g, ^c^25 g, ^d^5 g pack size.

**Scheme 1:**
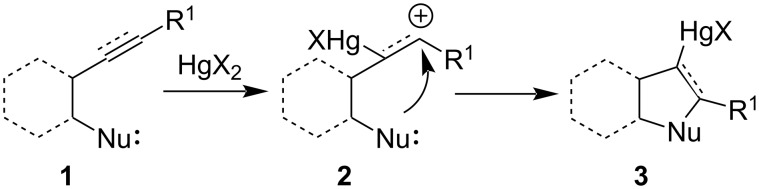
Schematic representation of Hg(II)-mediated addition to an unsaturated bond.

The generalized mechanism for cyclization reactions are, alkenes/alkynes **1** initially react with Hg(II) salts (HgX_2_) leading to the formation of a mercurial carbonium ion **2** followed by the attack of an intramolecular nucleophile giving rise to a cyclized mercuro–halide complex compound **3** (Scheme 1).

## Review

### Cyclization reactions involving stoichiometric amounts of Hg(II) salts

#### Cyclization involving alkenes (>C=C<)

**Scheme 2:**
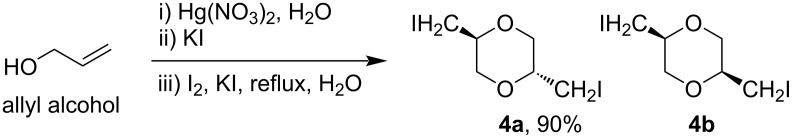
First report of Hg(II)-mediated synthesis of 2,5-dioxane derivatives from allyl alcohol.

In 1900 and 1901, the Sand and Biilmann group had first reported the Hg(II)-mediated cyclization of allyl alcohol using Hg(II) nitrate (Hg(NO_3_)_2_) in two separate publications [36–37]. The cyclized product thus formed was further treated with iodine to get *trans*-2,5-bis(iodomethyl)-*p*-dioxane (**4a**). The formation of *trans*-isomer **4a** as the major product and *cis*-isomer **4b** as a minor product was later confirmed by Summerbell et al. by repeating the same experiments (Scheme 2) [38].

**Scheme 3:**
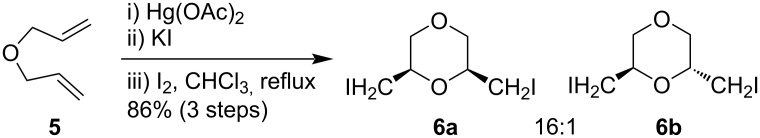
Stepwise synthesis of 2,6-distubstituted dioxane derivatives.

Cyclization of biallyl ether **5** in presence of mercuric acetate (Hg(OAc)_2_) in an aqueous medium was reported to synthesize a diastereomeric mixture of 2,6-disubstituted-*p*-dioxane **6**. The outcome of the reaction was much generalized with no detailed discussion about the ratio of diastereomeric products [39]. Later, Summerbell and co-workers modified the reaction conditions to synthesize 2,6-disubstituted dioxane derivatives **6** (*cis*/*trans* 16:1). Unlike 2,5-disubstituted dioxane derivatives, here the *cis*-isomer was the major product. A higher ratio of the *cis*-dioxane **6** was achieved by increasing the reaction time and acidity of the reaction medium, while elevated temperature showed no effect (Scheme 3) [40].

**Scheme 4:**
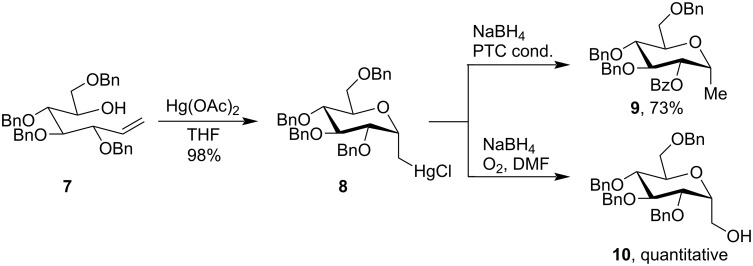
Cyclization of carbohydrate alkene precursor.

The mercuricyclization was also employed in the field of carbohydrate chemistry for the synthesis of α-ᴅ-*C*-glycopyranosyl derivatives. The reaction between carbohydrate alkene precursor **7** and Hg(OAc)_2_ proceeds with high stereoselectivity to give the α-ᴅ-*C*-glycopyranosyl derivative (1,5-*trans*-isomer) **8** as a single isomer [41]. Treatment of compound **8** with sodium borohydride (NaBH_4_) under phase transfer conditions (PTC) yields compound **9** as the only product. The selectivity of α-stereochemistry was primarily due to the strong directing effect of the neighboring benzyl ether group with the Hg(OAc)_2_. When cyclic mercuric halide **8** was treated with NaBH_4_ and oxygen (O_2_) in DMF oxidative demercuration takes place to give alcohol **10** in quantitative yield (Scheme 4).

**Scheme 5:**
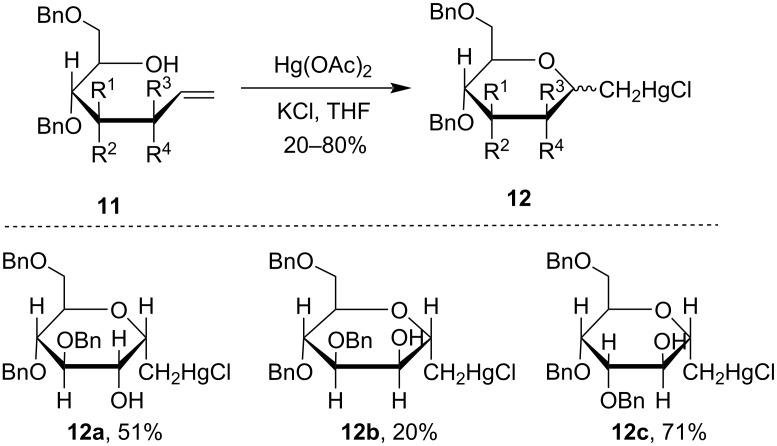
Hg(II)-mediated synthesis of *C*-glucopyranosyl derivatives.

The methodology utilized in Scheme 5 had been successfully employed for the preparation of *C*-glycopyranosyl derivatives of common and uncommon sugars like α-gluco, α-manno, β-altro, β-ido, α- and β-gulo, β-talo, α-galacto, and α-allo *C*-glycopyranosides [42]. Vinylated derivatives of aldopentoses **11** were treated with Hg(OAc)_2_ affording the corresponding cyclized pyanosylmethylmercuric chloride derivatives **12** (Scheme 5). This methodology can be used to synthesize rare *C*-glycosyl carbohydrates from easily available sugars.

**Scheme 6:**
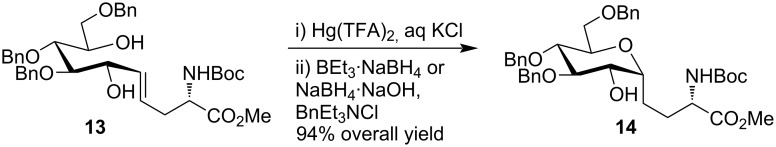
Synthesis of *C*-glycosyl amino acid derivative using Hg(TFA)_2_.

In a similar manner, stereoselective cyclization of *C*-glycosyl amino acid derivative **13** using mercuric trifluoroacetate Hg(TFA)_2_ at room temperature was performed. The reaction proceeds primarily through stereoselective cyclization to give α-ᴅ-*C*-glycopyranosyl amino acid derivative **14** as the major product [43]. Nevertheless, *C*-mannopyranosyl derivatives cannot be achieved in a similar manner as reductive elimination forms during the mercury removal process (Scheme 6).

**Scheme 7:**
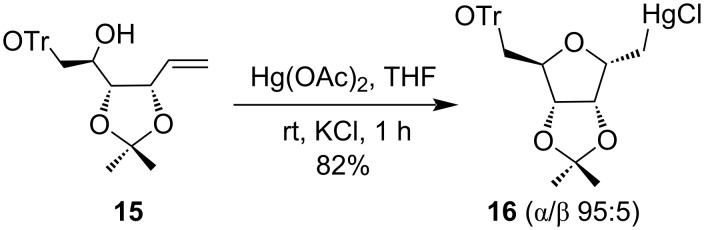
Hg(OAc)_2_-mediated synthesis of α-ᴅ-ribose derivative.

Mercury(II) salts had been effectively used to synthesize five-membered furanose derivatives with high stereoselectivity. Nicotra et al. developed Hg(OAc)_2_-mediated cyclization of hydroxy-alkene derivative **15** to form α-ᴅ-ribose derivative **16** at room temperature (Scheme 7). They had confirmed the formation of the α-isomer of ᴅ-*C*-ribofuranosyl **16** predominantly over the β-isomer (α/β 95:5) [44].

**Scheme 8:**
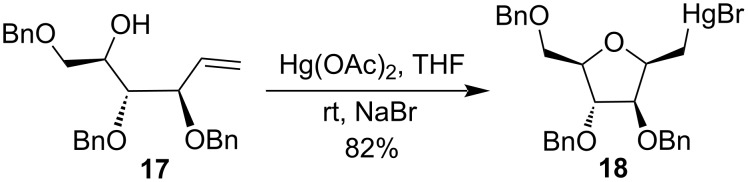
Synthesis of β-ᴅ-arabinose derivative **18**.

In contrast, when Reitz and co-workers carried out a ring formation of benzylated *C*-arabinofuranoside derivative **17** in presence of 1.4 equiv of Hg(OAc)_2_ and sodium bromide (NaBr) at room temperature, then β-ᴅ-arabinose derivative **18** was formed as the major product (Scheme 8) [45]. The high stereoselectivity of β-derivative **18** at the anomeric position was predominantly due to the presence of the benzyl groups at the C-2 and C-3 positions of the starting material.

It had been cited in many publications that the stereoselectivity of products formed due to Hg(II)-salt-mediated cyclization reactions of alkene-alcohol derivatives depends on several factors: the nature of the Hg(II) salts [46], the starting materials [47], and the effect of H_2_O (protic solvent) in the reaction media [48].

**Scheme 9:**
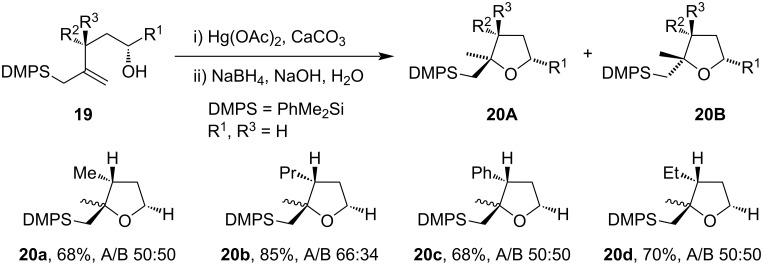
Hg(OAc)_2_-mediated synthesis of tetrahydrofuran derivatives.

Pulido et al. had developed a conversion of allylsilyl alcohols **19** to diastereomeric mixtures of tetrahydrofuran derivatives **20A** and **20B** (Scheme 9) [47]. It was reported that more substituted alkyl groups present in allylsilyl alcohol **20b** direct the selective formation of diastereomeric products. They also observed that changes in Hg(II) salts result in different ratios of the *cis*- and *trans*-isomer in the cyclized products [47]. The differences in *cis* and *trans* ratios were primarily due to the basicity of anions associated with Hg(II) salts [46].

**Scheme 10:**
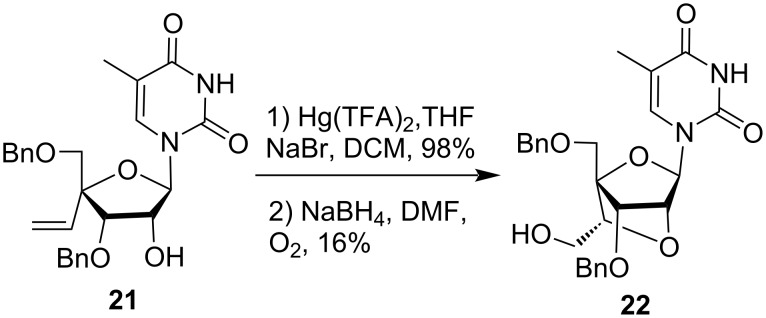
Synthesis of Hg(TFA)_2_-mediated bicyclic nucleoside derivative.

A similar type of reaction methodology was employed for the formation of a bicyclic nucleoside analog. 4'-*C*-vinylribofuranoside derivative **21** on treatment with Hg(TFA)_2_ followed by reduction with NaBH_4_ leads to the formation of bicyclic nucleoside derivative **22** (Scheme 10) [49].

**Scheme 11:**
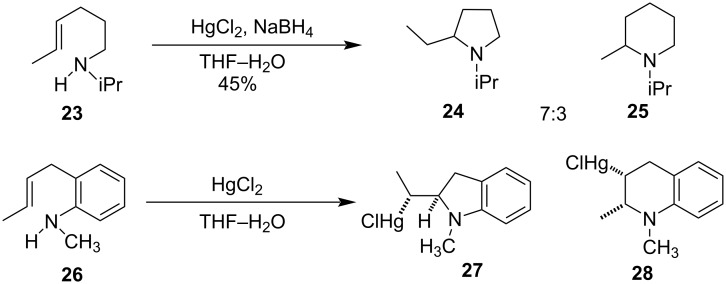
Synthesis of pyrrolidine and piperidine derivatives.

Pyrrolidine and piperidine derivatives were also synthesized by Hg(II)-salt-induced cyclization. *N*-Isopropyl-1-aminohex-4-ene (**23**) on treatment with 1 equiv HgCl_2_ followed by reduction with NaBH_4_ yielded pyrrolidine **24** and piperidine derivative **25** in the ratio of 7:3 [50]. *N*-Methylaniline derivative **26** undergoes cyclization with HgCl_2_ and gave *cis*-mercuro chlorides **27** and **28** as isolated products (Scheme 11) [51].

**Scheme 12:**
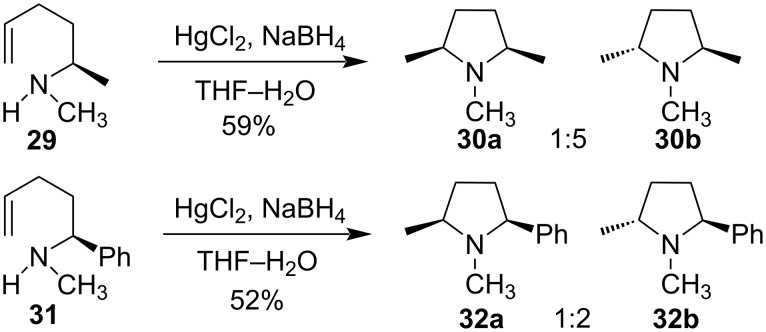
HgCl_2_-mediated synthesis of diastereomeric pyrrolidine derivatives.

For the synthesis of diastereomeric pyrrolidine derivatives, a Hg(II) salt had been used. When HgCl_2_ was added to secondary methylamine derivatives **29** and **31** followed by reduction with NaBH_4_ a mixture of diastereomers **30a**,**b** and **32a**,**b** was obtained, respectively. *trans*-Isomers were formed as major products over *cis*-isomers (Scheme 12) [52].

**Scheme 13:**
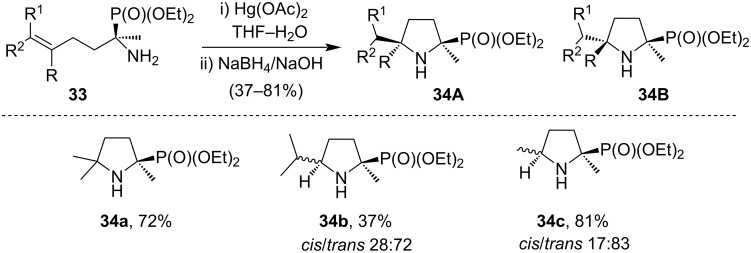
HgCl_2_-mediated cyclization of alkenyl α-aminophosphonates.

Five- and six-membered *N*-containing heterocyclic phosphonates were synthesized by intramolecular cyclization of alkenyl α-aminophosphonates in a similar way with the treatment of Hg(OAc)_2_ [53]. The cyclized products **34A**,**B** formed from starting material **33** were regiospecific and followed Markovnikoff’s type addition in the reaction [54–57]. It was also reported that the formation of α-phosphorylated pyrrolidines mostly takes place in regio- and stereoselective ways depending on the reaction conditions (Scheme 13) [53,58].

**Scheme 14:**
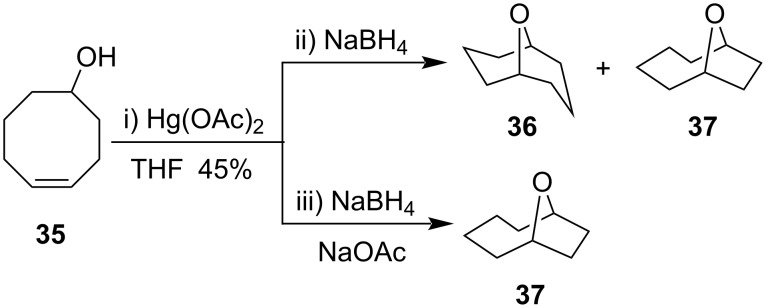
Cyclization of 4-cycloocten-1-ol with Hg(OAc)_2_ forming fused bicyclic products.

Cyclization of 4-cycloocten-1-ol (**35**) with Hg(OAc)_2_ resulted in formation of two types of fused bicyclic products, 9-oxabicyclo[3.3.1]nonane (**36)** and 9-oxabicyclo[4.2.1]nonane (**37**) (Scheme 14) [59–60]. It was observed that the regioselective synthesis of 9-oxabicyclo[4.2.1]nonane (**37**) was favored when NaBH_4_ and sodium acetate NaOAc were present, while in absence of NaOAc, 9-oxabicyclo[3.3.1]nonane (**36)** was formed as an exclusive product.

**Scheme 15:**
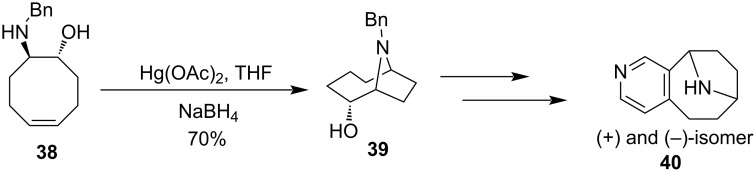
*trans*-Amino alcohol formation through Hg(II)-salt-mediated cyclization.

In a similar manner, an aminomercuration reaction had been successfully employed in the cyclization of *trans*-amino alcohol **38** leading to the formation of (1*R*,2*R*,6*R*)-9-benzyl-9-azabicyclo[4.2.1]nonan-2-ol (**39**). The bicyclic derivative **39** through the number of consequent reactions formed a highly potent (+)/(−)-pyrido[3,4-*b*]homotropane (**40**), a bridged nicotinoid (Scheme 15) [61].

**Scheme 16:**
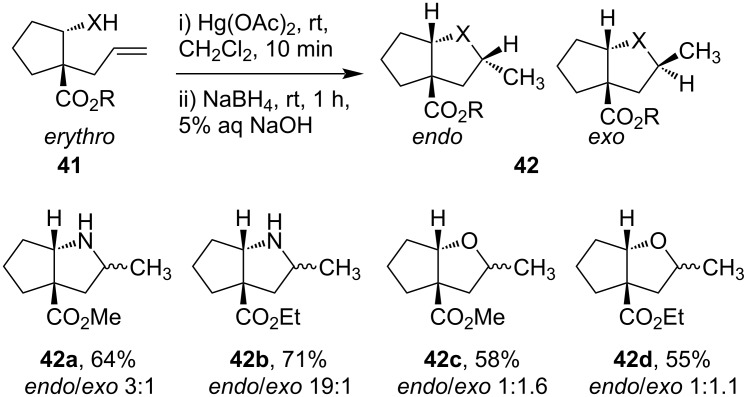
Hg(OAc)_2_-mediated 2-aza- or 2-oxa-bicyclic ring formations.

Similarly, precursor **41** at room temperature in the presence of Hg(OAc)_2_ (1 equiv) in CH_2_Cl_2_ cyclized to form 2-aza- or 2-oxabicyclic mercuri derivatives. Further, treatment of intermediates with NaBH_4_ (4 equiv) in presence of 5% aq NaOH solution for 1 hour at room temperature produced bicyclic derivatives **42** (Scheme 16) [62]. The diasteroselectivity of the products formed depends on the heteroatoms involved; azamercuration yields more selectivity than oxymercuration.

**Scheme 17:**
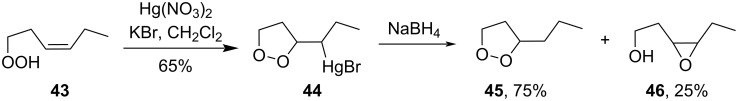
Hg(II)-salt-induced cyclic peroxide formation.

Nixon et al. reported a Hg(II)-salt-induced alkenyl hydroperoxide **43** cyclization to synthesize cyclic peroxides **44** as the major product, which on further treatment with NaBH_4_ gives compound **45** along with the unexpected product **46** (Scheme 17) [63].

**Scheme 18:**
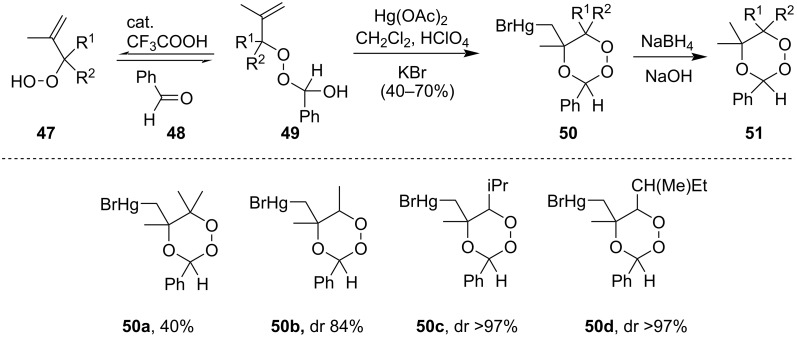
Hg(OAc)_2_-mediated formation of 1,2,4-trioxanes.

Later they had reported the reaction between alkenyl hydroperoxide **47** and aldehydes **48** to form hemiperoxyacetal **49** which subsequently reacted with Hg(OAc)_2_ and a catalytic amount of perchloric acid (HClO_4_) forming cyclized product 1,2,4-trioxanemercuri bromide **50**. The product thus formed was reduced with NaBH_4_ to form compound **51** (Scheme 18) [64].

**Scheme 19:**
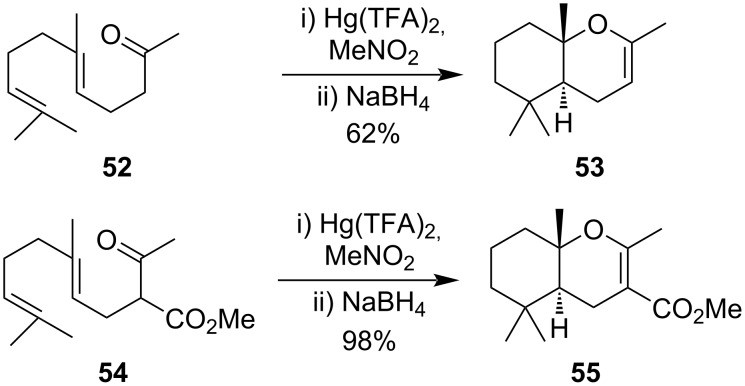
Endocyclic enol ether derivative formation through Hg(II) salts.

Kurbanov et al. first reported the cyclization of different isoprenoid derivatives to hexahydrochromene derivatives using Hg(II) salts [65]. They had shown that *trans*-geranylacetone (**52**) produces *trans*-2,5,5,9-tetramethylhexahydrochromene (**53**) while the *cis-*cyclized product was formed from *cis*-isoprenoid derivatives. Later, Hoye et al. used similar reaction conditions to cyclize dienes **54** with 1.1 equiv of Hg(TFA)_2_ to yield endocyclic enol ether **55** in almost quantitative yield [66]. They had also performed experiments with different isoprenoid derivatives (carboxylic acid, ketones alcohols, and keto esters) to form different bicyclic products (Scheme 19).

**Scheme 20:**
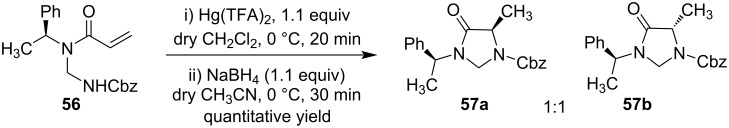
Synthesis of optically active cyclic alanine derivatives.

A Hg(II)-salt-promoted cyclization of amidal **56** was performed to synthesize optically active cyclic alanine derivatives **57** as 1:1 diastereomeric mixture [67]. Diastereomers were further separated by column chromatography and afforded enantiomerically pure isomers of *N*-substituted imidazolidine-4-ones **57** (Scheme 20).

**Scheme 21:**
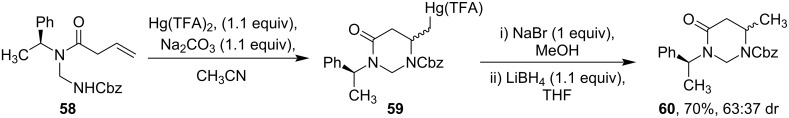
Hg(II)-salt-mediated formation of tetrahydropyrimidin-4(1*H*)-one derivatives.

In a similar manner, *N*-Cbz-protected amine **58** undergoes cyclization using Hg(TFA)_2_ (1 equiv) and yielded a dinitrogen-containing mixture of diastereomeric alicyclic derivatives **59**. The tetrahydropyrimidin-4(1*H*)-one-mercuri trifluoroacetate derivative **59** on successive treatment with NaBr and LiBH_4_ gives a mixture of tetrahydropyrimidin-4(1*H*)-one derivatives **60** in the diastereomeric ratio of 63:37 after separation by column chromatography [68] (Scheme 21).

**Scheme 22:**
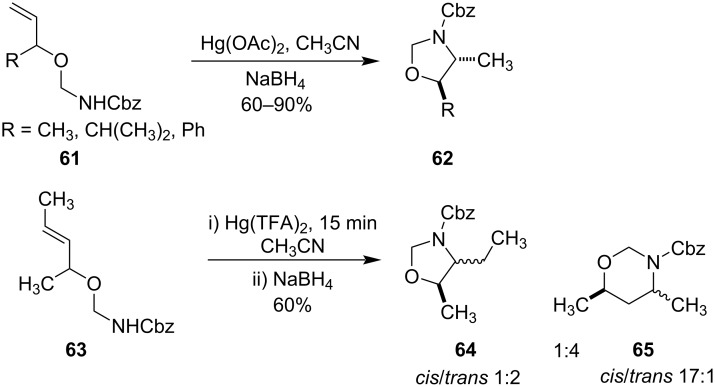
Cyclization of ether derivatives to form stereoselective oxazolidine derivatives.

Mercury(II) salts had also been successfully utilized in the cyclization of ether derivatives **61** to form stereoselectively *trans*-4,5-disubstituted oxazolidine derivatives **62** (Scheme 22) [69]. Later it was reported, when homologous allyloxycarbamate derivative **63** was cyclized with Hg(TFA)_2_ then five- and six-membered rings **64** and **65** were formed with a 1:4 ratio (Scheme 22) [70]. The greater yield of the six-membered product was primarily due to the electron-withdrawing effect of ethereal oxygen which in turn destabilizes the carbocation at the β-carbon and hence the nucleophilic attack at the γ-carbon took place.

**Scheme 23:**
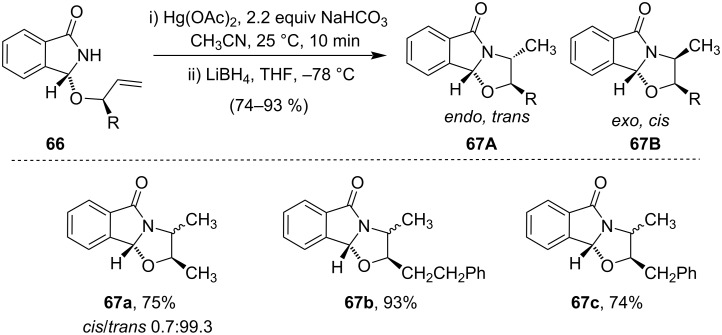
Cyclization of amide derivatives induced by Hg(OAc)_2_.

Cyclization of amide derivative **66** induced by Hg(OAc)_2_ followed by reduction with LiBH_4_ afforded a mixture of compounds **67A**/**67B** [71]. The formation of *endo*,*trans*-product as a major product over the *exo*,*cis*-isomer was primarily due to a stereoinduction effect (Scheme 23). The small amount of *exo,cis*-isomers **67Ba/67Bc** was only detected by HPLC analysis of the crude reaction mixture.

**Scheme 24:**
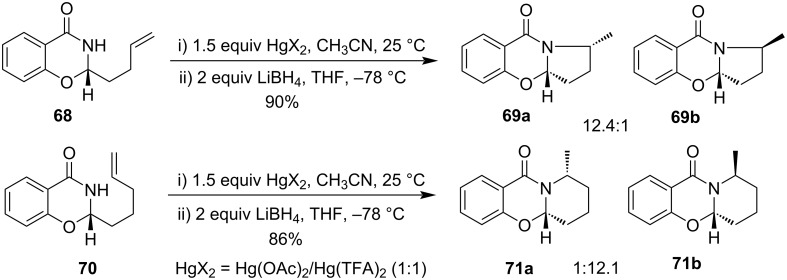
Hg(OAc)_2_/Hg(TFA)_2_-promoted cyclization of salicylamide-derived amidal auxiliary derivatives.

Takacs et al. observed the rapid cyclization of salicylamide-derived amidal auxiliary derivatives **68** and **70** in presence of 1.5 equiv of a 1:1 mixture of Hg(OAc)_2_/Hg(TFA)_2_ resulting in a diastereomeric pair of cyclized products **69** and **71**, respectively. It was observed that the *cis*-isomer was predominant in the case of the five-membered ring while the *trans*-isomer was predominant in the case of the six-membered ring formation (Scheme 24) [72].

#### Cyclization involving alkynes (-C≡C-)

**Scheme 25:**
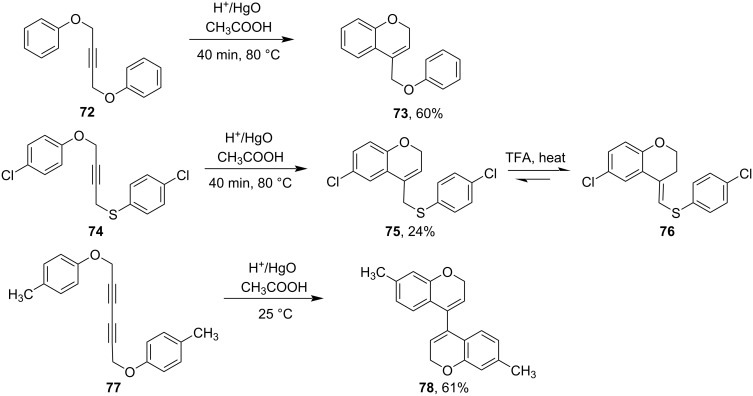
Hg(II)-salt-mediated cyclization to form dihydrobenzopyrans.

Hg(II)-salt-mediated cyclizations were also observed in the case of alkynes. Arylalkynes **72** were cyclized via Hg(II)-salt-induced reactions to form benzopyran derivatives **73** [73–75]. Interestingly in the case of 1-aryloxy-4-arylthio-2-butyne derivatives **74**, selective ring closure on the oxygen side afforded 6-membered chromene derivatives **75** but no thiochromene derivatives were observed [76]. Compound **75** on refluxing with trifluoroacetic acid isomerized to form exocyclic *trans*-compound **76**. It was reported that 1,4-di(arylthiol)-2-butyne derivatives only afforded usual Hg(II)-salt-mediated hydrated products instead of cyclized thiochromene derivatives. Balasubramanian et al. had performed a mercuric-oxide-mediated cyclization of 1,6-di(aryloxy)-2,4-hexadiyne derivatives **77** to get bichromene derivatives **78** (Scheme 25) [77].

**Scheme 26:**
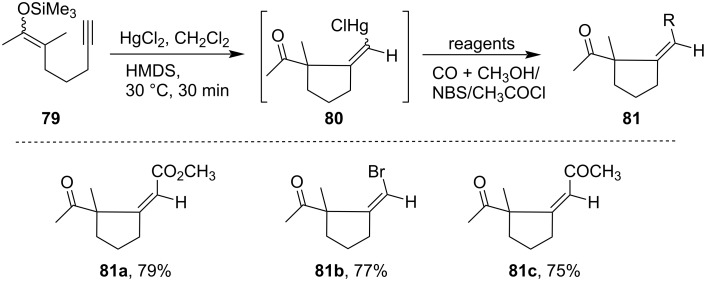
HgCl_2_-induced cyclization of acetylenic silyl enol ether derivatives.

A HgCl_2_-induced cyclization also takes place for acetylenic silyl enol ether derivative **79** forming carbocyclic compounds **81** with good yields via intermediate **80** [78]. The cyclization of compounds **79** undergo regioselective addition with the triple bond in exocyclic alkene position leading to the formation of α-mercury ketone **80**, which were later functionalized by electrophilic addition (Scheme 26).

**Scheme 27:**
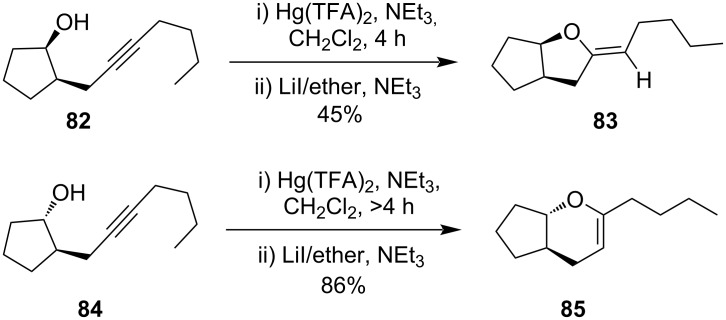
Synthesis of exocyclic and endocyclic enol ether derivatives.

Schwartz et al. developed a new route for the synthesis of exocyclic enol ether **83** and endocyclic enol ether **85** involving a Hg(II)-induced cyclization of acetylenic alcohols **82** and **84**, respectively [79]. This work revealed that the regioselectivity of the Hg(II) addition to the alkyne may be influenced by diastereomers; in the case of *cis*-isomer **82** exocyclic enol ether **83** was formed while for *trans*-isomer **84** the reaction takes place with a much slower rate yielding endocyclic enol ether **85** as the only product (Scheme 27) [79].

**Scheme 28:**
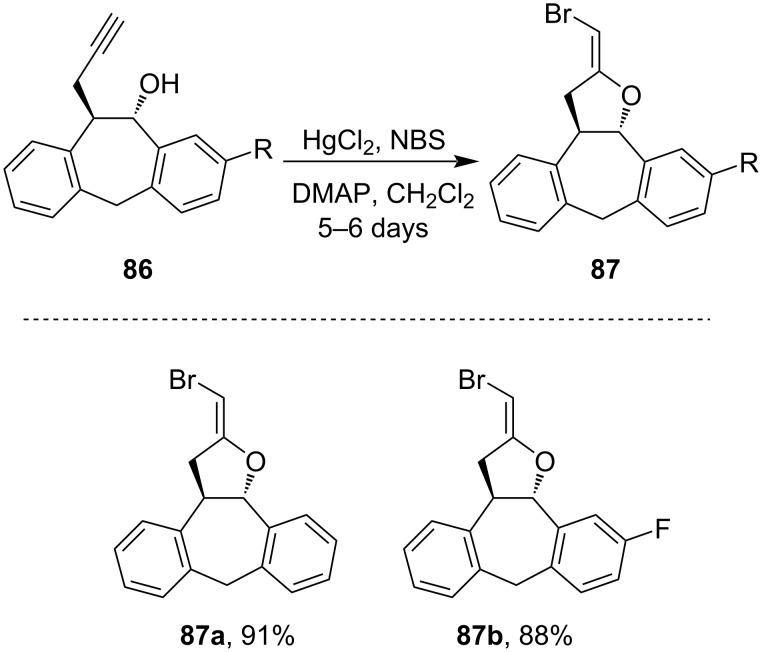
Cyclization of *trans-*acetylenic alcohol by treatment with HgCl_2_.

*trans-*Acetylenic alcohol **86** on treatment with HgCl_2_ (0.5 equiv) in presence of *N*-bromosuccinimide (NBS) undergoes cyclization yielding stable bromo alkenes **87** (Scheme 28) [80–81].

**Scheme 29:**
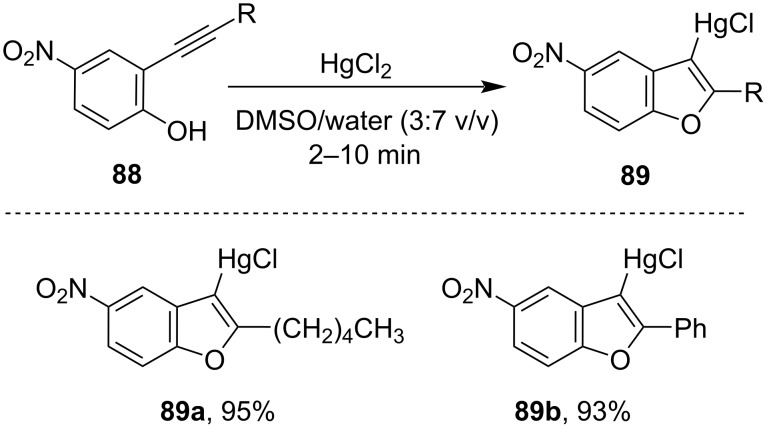
Synthesis of benzofuran derivatives in presence of HgCl_2_.

Atta et al. reported the specific cyclization of ethynyl phenols **88** in presence of HgCl_2_ at ambient temperature yielding benzofuran derivatives **89**. They had reported that the electron-withdrawing electronic effect at the aromatic ring promotes the cyclization reaction, whereas there is no cyclization in the absence of the withdrawing group (Scheme 29) [82].

**Scheme 30:**
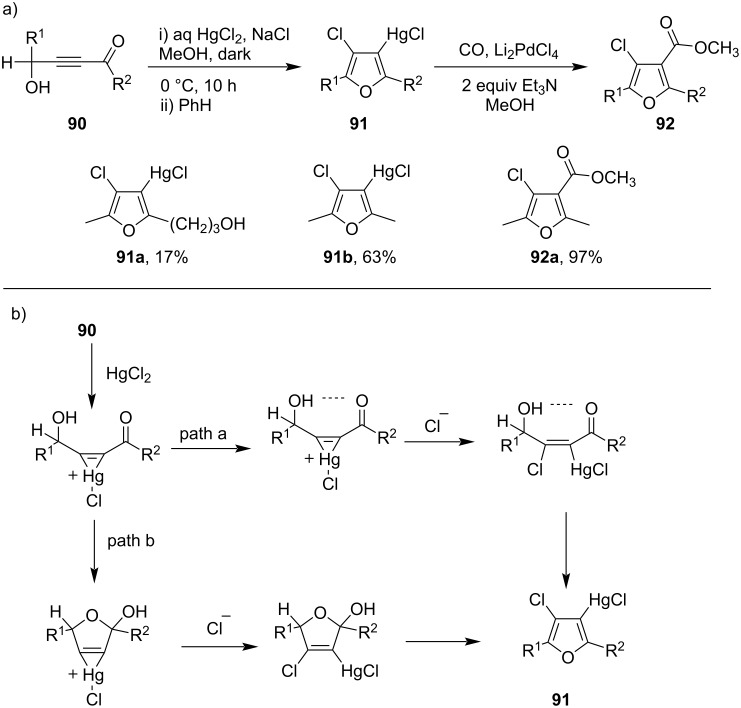
a) Hg(II)-salt-mediated cyclization of 4-hydroxy-2-alkyn-1-ones to furan derivatives and b) its mechanistic pathway.

Larock et al. had performed the mercuration of 4-hydroxy-2-alkyn-1-ones **90** with HgCl_2_ to get furylmercurials **91** via *syn*-addition of acetylene, which on carbonylation yielded the furan-containing carbonyl compound **92** (Scheme 30) [83]. It was proposed that initially mercuration of acetylene bonds via mercurinium like ions or π-complex takes place. Then the structure was stabilized through hydrogen bonding between alcohol and carbonyl groups (path a) or by hemiketal formation (path b). Thus, the chloride ion attacks from the frontside, producing *syn*-addition molecules that, upon dehydration, formed furan rings.

**Scheme 31:**
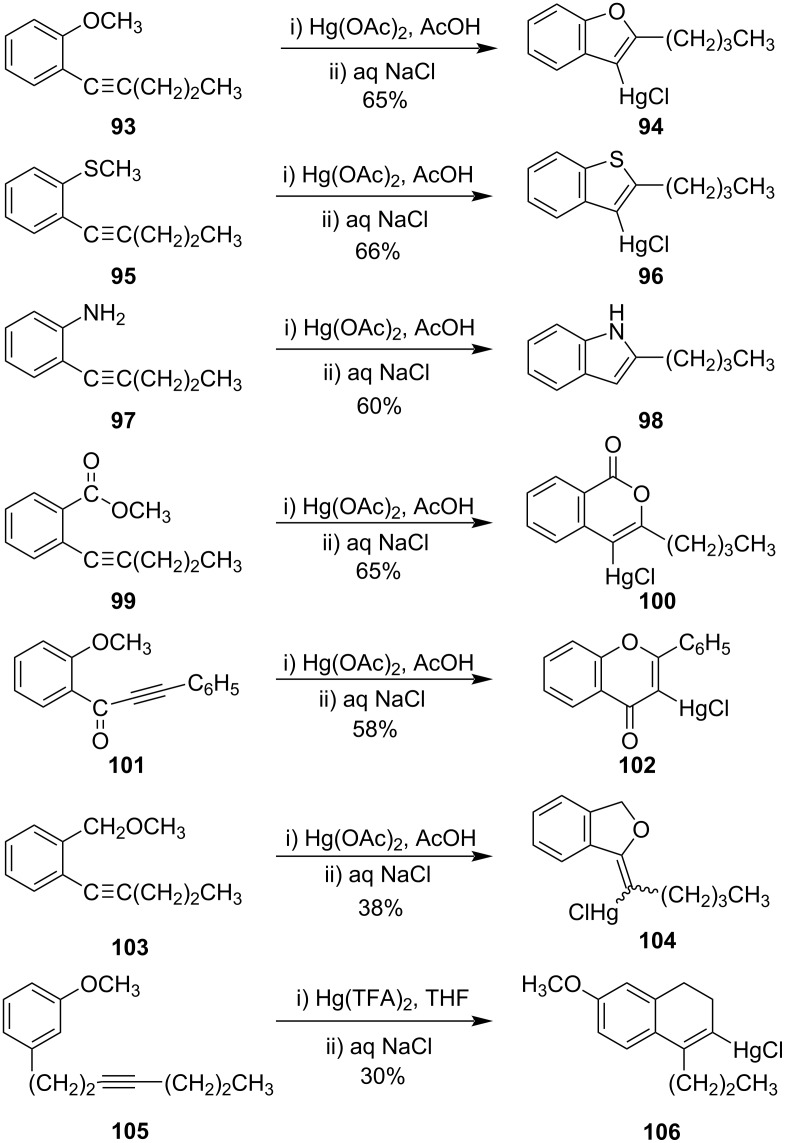
Cyclization of arylacetylenes to synthesize carbocyclic and heterocyclic derivatives.

Later they had reported similar mercuration of arylacetylenes to synthesize a broad spectrum of heterocycles, namely benzofurans, benzothiophenes, isocoumarins, chromones, benzopyrans, 1,2-dihydronaphthaIenes, coumarins, and coumestan including some physiologically active heterocyclic natural products like flavones [84]. In the presence of Hg(OAc)_2_ in acetic acid, simple cyclization of *ortho*-substituted arylacetylenes **93**, **95**, and **97** yielded benzofurans **94**, benzothiophenes **96**, and indoles **98**, respectively. When the carbonyl group was introduced between an aryl and a methoxy group (**99**) then six-membered isocoumarin ring **100** was formed, and when a carbonyl group was introduced in between an aryl and an alkyne group (**101**), chromone derivatives **102** were formed. On the introduction of the methylene moiety in between the aryl and methoxy group, **103** yielded isofuran derivatives **104** due to the change in regioselectivity during the cyclization. Dihydronapthalenes **106** were synthesized by cyclization of the corresponding methoxyarylalkyne derivative **105** (Scheme 31).

**Scheme 32:**
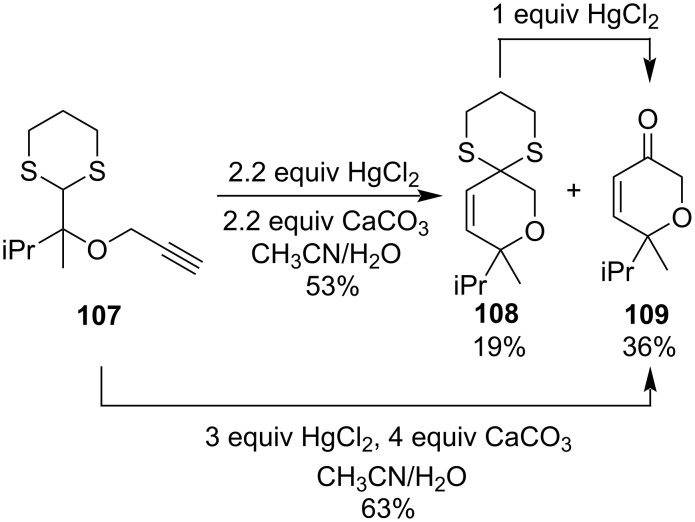
Hg(II)-salt-promoted cyclization–rearrangement to form heterocyclic compounds.

In 2005, Ghorai et al. reported the synthesis of a mixture of 6-membered as well as 5-membered heterocyclic compounds through a Hg(II)-salt-promoted unique cyclization–rearrangement reaction [85]. They had performed the HgCl_2_-promoted cyclization of *O*-propargyl glycolaldehyde dithioacetals **107** via their dithioketals and dithioacetals to synthesize six-membered heterocycles **108** and **109** (Scheme 32). Five-membered dihydrofuryl aldehydes were also isolated as minor products in some examples.

**Scheme 33:**
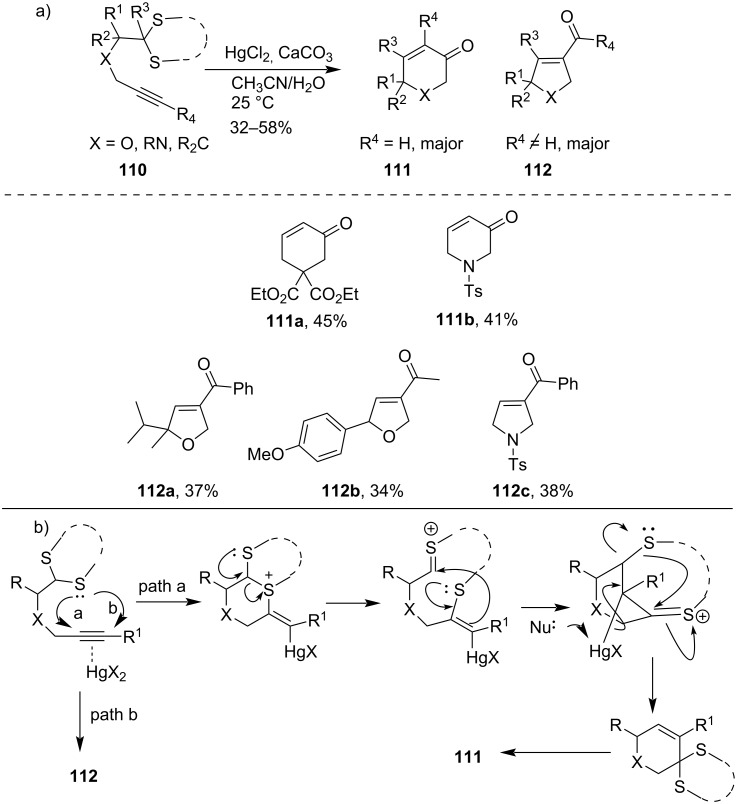
a) HgCl_2_-mediated cyclization reaction of tethered alkyne dithioacetals; and b) proposed mechanism.

Following a similar protocol, Biswas et al. later published the HgCl_2_-mediated cyclization reaction of tethered alkynedithioacetals **110** to provide six- and five-membered carbocyclic and heterocyclic derivatives **111** and **112**, respectively. They had observed that the formation of five-membered rings (**112a**–**c**) was preferred when substitutents were present at the alkyne terminus, whereas six-membered rings (**111a**,**b**) formed predominantly in case of unsubstituted alkyne dithioacetals (Scheme 33) [86]. They had reported the plausible mechanism for the formation of a six-membered pyranose ring follows ‘path a,' while for the formation of five-membered pyrrolidine derivatives ‘path b' was followed.

#### Cyclization involving allenes (>C=C=C<)

Apart from alkenes/alkynes, there are also examples where cyclization takes place involving allene functionalities. Some of the examples are discussed below.

**Scheme 34:**
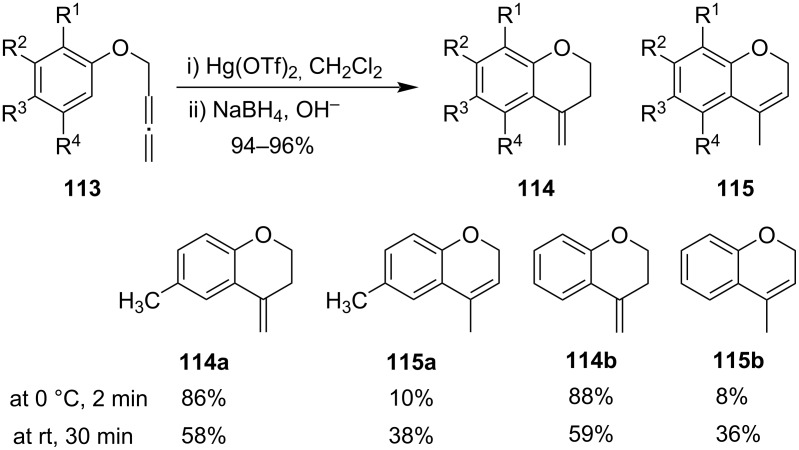
Cyclization of aryl allenic ethers on treatment with Hg(OTf)_2_.

Balasubramanian et al. reported the cyclization of aryl allenic ethers **113** on treatment with Hg(OTf)_2_. Compound **113** reacted with 1.1 equiv of Hg(OTf)_2_ at room temperature followed by reduction with alkaline NaBH_4_ leading to the formation of benzopyran derivatives **114** and **115**. The ratio of the formation of products depends on the temperature and time of the reaction (Scheme 34) [87].

**Scheme 35:**
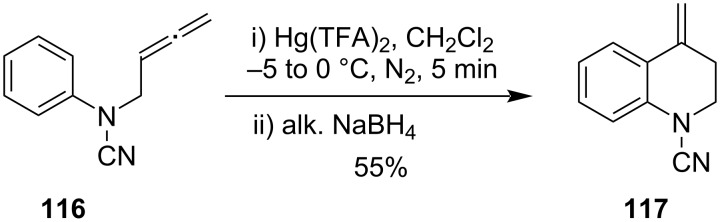
Hg(TFA)_2_-mediated cyclization of allene.

Devan et al. had developed similar types of Hg(TFA)_2_-mediated cyclizations of allene **116** at low temperature followed by reduction with alkaline NaBH_4_ to form cyclized product **117** in moderate yield [88]. The reaction was believed to proceed through Hg(II) ion-promoted electrophilic cyclization (Scheme 35).

### Cyclization involving catalytic Hg(II) salts

#### Cyclization involving alkenes (>C=C<)

Apart from the stoichiometric amount used in cyclization, there is abundant literature where a catalytic amount of Hg(II) salt was employed for cyclization reactions between nucleophiles and unsaturated bonds.

**Scheme 36:**
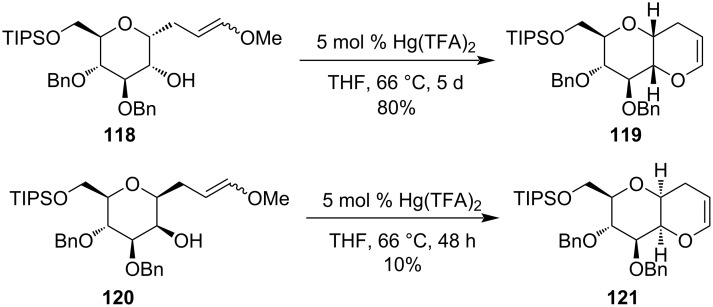
Hg(II)-catalyzed intramolecular *trans*-etherification reaction of 2-hydroxy-1-(γ-methoxyallyl)tetrahydropyran derivatives.

The synthesis of fused polycyclic ethers by the treatment of a catalytic amount of Hg(TFA)_2_ with suitable starting material was demonstrated by Tan et al. [89]. They had reported a Hg(II)-catalyzed intramolecular *trans*-etherification reaction of 2-hydroxy-1-(γ-methoxyallyl)tetrahydropyrans **118** and **120** to the corresponding bicyclic dihydropyrans **119** and **121**, respectively (Scheme 36).

**Scheme 37:**
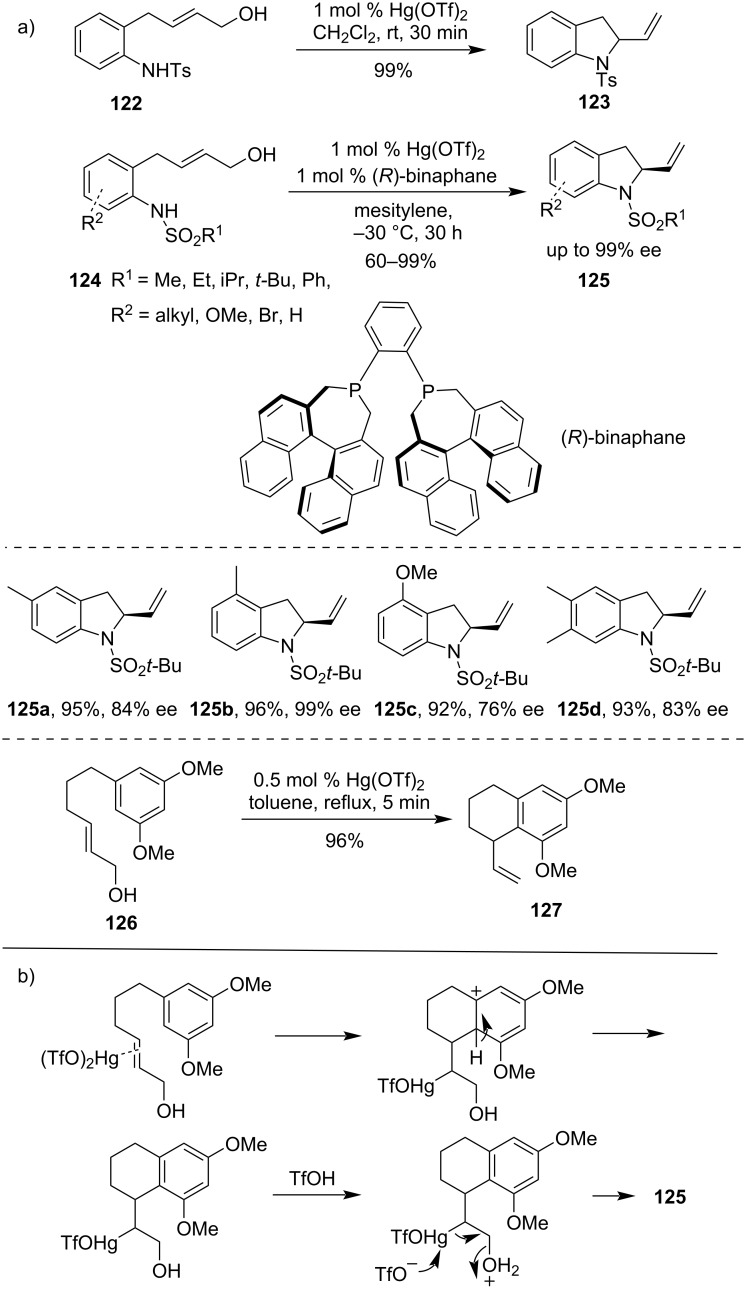
a) Cyclization of alkene derivatives by catalytic Hg(OTf)_2_ salts and b) mechanism of cyclization.

Later, Namba et al. reported the synthesis of racemic vinylindoline derivatives **123** from *N*-tosylanilinoallylic alcohol derivative **122** by using 1–2 mol % of Hg(OTf)_2_ in CH_2_Cl_2_ at room temperature [90]. An asymmetric synthesis of vinylindoline derivatives **125** was achieved by utilizing chiral ligands like chiral binaphane (Scheme 37) [91]. They had observed that the formation of six- and seven-membered rings required elevated temperatures. Subsequently, the same group studied the cyclization of arylene **126** to furnish naphthalene derivative **127**. The plausible mechanism for the formation of compound **127** proceeded consecutively with π-complex formation, Friedel–Crafts type addition, deprotonation, and finally protonation of alcohol for the elimination of water to get the final product [92].

**Scheme 38:**
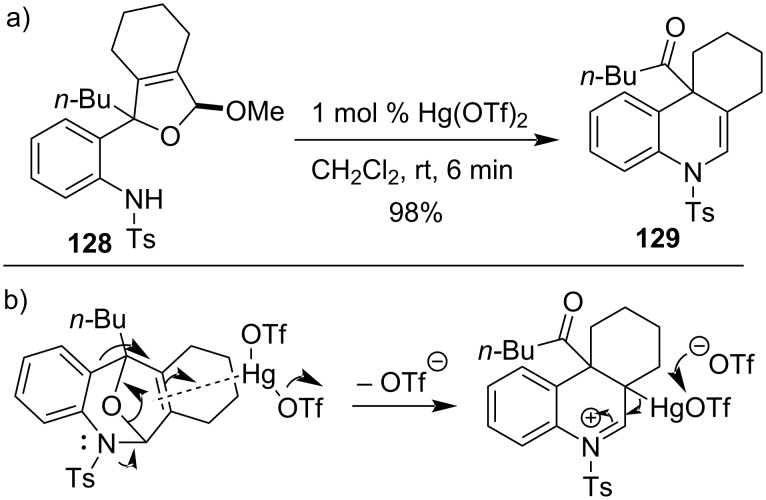
a) Synthesis of 1,4-dihydroquinoline derivatives by Hg(OTf)_2_ and b) plausible mechanism of formation.

A Hg(OTf)_2_-mediated cyclization was utilized for the synthesis of 1,4-dihydroquinoline **129** possessing a quaternary carbon center from **128** [93]. The reaction was reported via a seven-membered bicyclic hemiaminal as mentioned in the mechanism. This catalytic rearrangement protocol was successfully applied for the construction of complex carbon frameworks from various tosylanilinoallyl acetals. 4*H*-Chromene derivatives were also synthesized starting from phenol derivatives (Scheme 38).

#### Cyclization involving alkynes (-C≡C-)

**Scheme 39:**
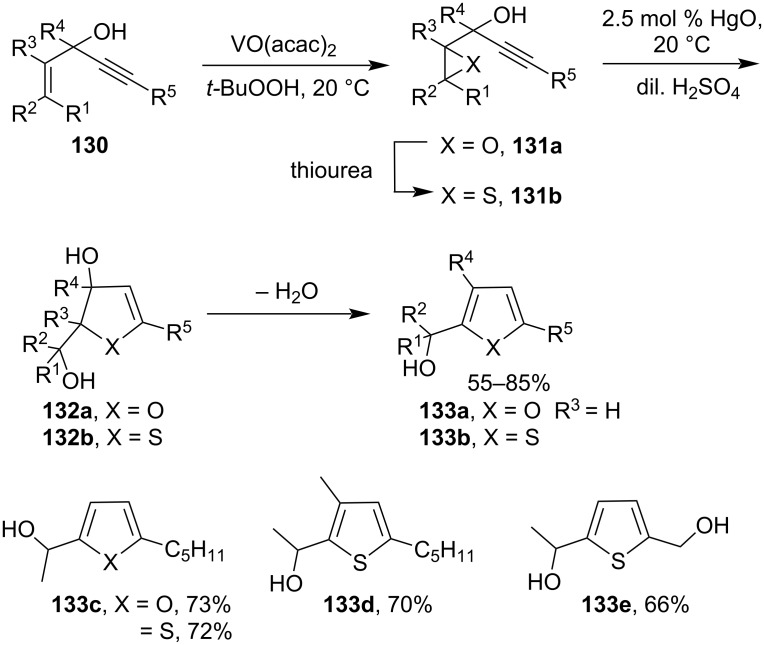
Synthesis of Hg(II)-salt-catalyzed heteroaromatic derivatives.

Marson et al. had developed the synthesis of substituted furans **133a–c** promoted by catalytic use of Hg(II) salt through cyclization of secondary and tertiary l-alkynyl-2,3-epoxyalcohols **131a** [94]. This is an example of a Hg(II)-salt-catalyzed rearrangement of 1-alkynyl-2,3-epoxy alcohols to substituted furans. The furan **133a** was formed by dehydration of intermediate **132a** through the corresponding oxonium cation. When R^3^ is an alkyl group, direct dehydration was not possible because of blocking. Later they reported a similar transformation of thiiranes to provide a wide variety of substituted thiophenes. They had synthesized substituted thiophenes **133b** starting from thiiranes **131b** via cyclization utilizing the catalytic amount of HgO and dil. H_2_SO_4_ at room temperature [95]. Initially, the Hg(II)-salt-catalyzed the formation of intermediate **132b** which further proceeded by dehydration to yield thiophenes **133b** as the final product (Scheme 39).

**Scheme 40:**
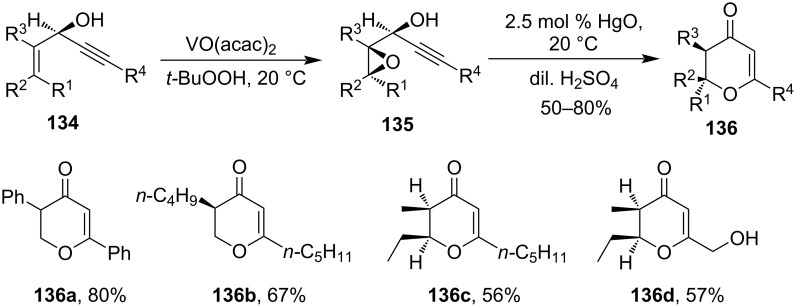
Hg(II)-salt-catalyzed synthesis of dihydropyranone derivatives.

Starting with 1-alkynyl-2,3-epoxy alcohols **135**, Marson et al. had reported a Hg(II)-salt-catalyzed rearrangement to produce 2,3-disubstituted-2,3-dihydropyranone derivatives **136**. The stereochemistry of substituents at 2,3-positions of 2,3-dihydropyranone **136** was controlled by *cis*- and *trans*-configuration of the epoxide of starting materials (Scheme 40) [96].

**Scheme 41:**
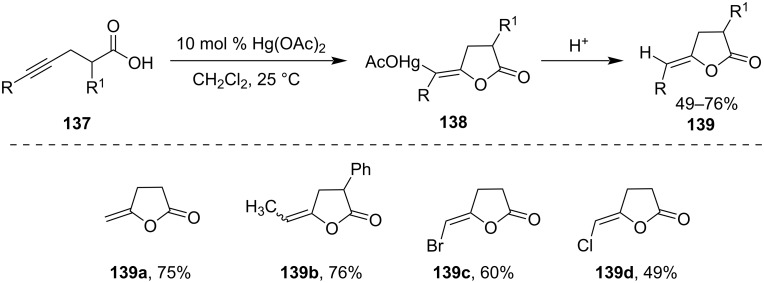
Hg(II)-salt-catalyzed cyclization of alkynoic acids.

Several unsaturated lactones had been synthesized from alkynoic acids via Hg(II)-salt-catalyzed cyclization reaction. For example, simple 4-pentynoic acid derivatives **137** afforded *γ*-methylene butyrolactones **139** in good yields via the formation of organomercural compounds **138** using catalytic mercuric oxide, mercuric acetate, or mercuric trifluoroacetate as shown in Scheme 41 [97].

**Scheme 42:**
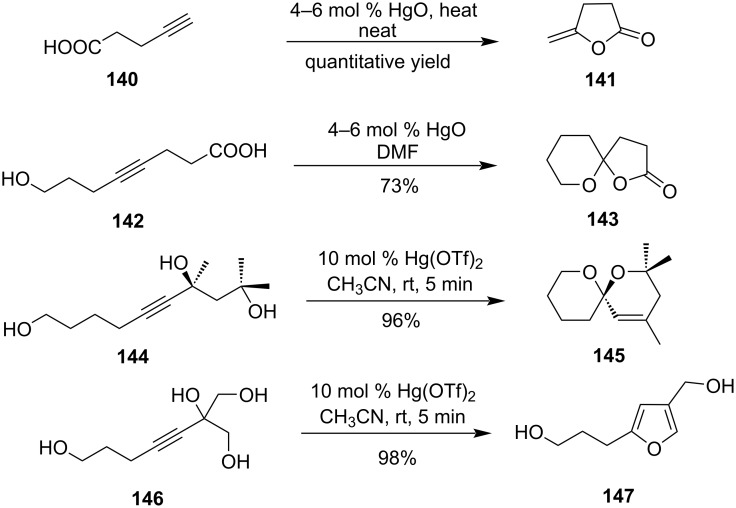
Hg(II)-salt-mediated cyclization of alkyne carboxylic acids and alcohol to furan, pyran, and spirocyclic derivatives.

Alkyne carboxylic acids also undergo oxymercuration reactions to form furan- and pyran-like derivatives. When γ-alkyne carboxylic derivative **140** was refluxed with HgO the cyclization took place to give product **141** in almost quantitative yields [98–99]. Compound **142** under refluxing conditions gave spirocyclic compound **143** as the exclusive product (Scheme 42) [99]. Propargylic triols **144** undergo Hg(OTf)_2_-catalyzed cyclization reaction, to produce monounsaturated spiroketal **145**. Finally in the case of 1,2-dihydroxy-3-alkynes **146** comparable reaction conditions leads to the formation of 2.5-disubstitued-furan **147** rather than mono-unsaturated spiroketals [100].

**Scheme 43:**
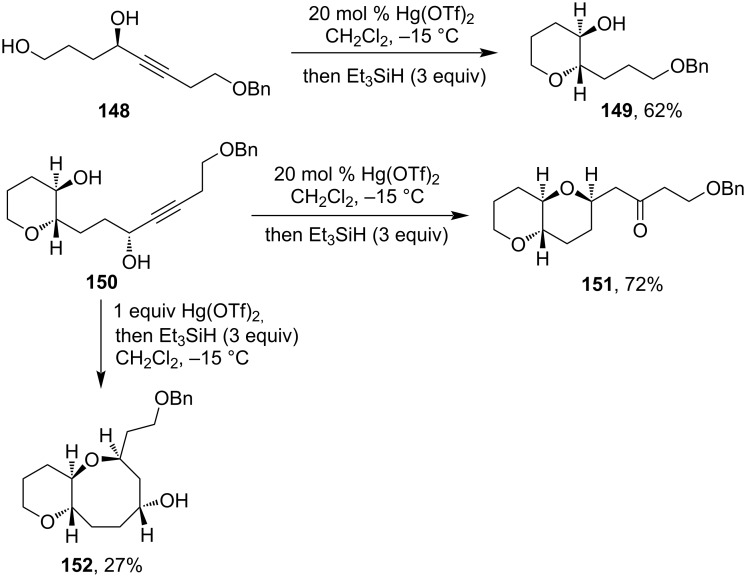
Hg(II)-salt-mediated cyclization of 1,4-dihydroxy-5-alkyne derivatives.

Interestingly when 1.4-dihydroxy-5-alkyne derivatives were subjected to a Hg(OTf)_2_-catalyzed cyclization then oxacyclization takes place to form tetrahydropyran derivatives (Scheme 43) [101]. Later it was shown that alkynyl diol **150** when treated with 20 mol % Hg(OTf)_2_ followed by Et_3_SiH afforded bispyranoyl ketone **151**, but when Hg(OTf)_2_ was increased (1 equiv) then fused pyran-oxocane derivative **152** was isolated [102].

**Scheme 44:**
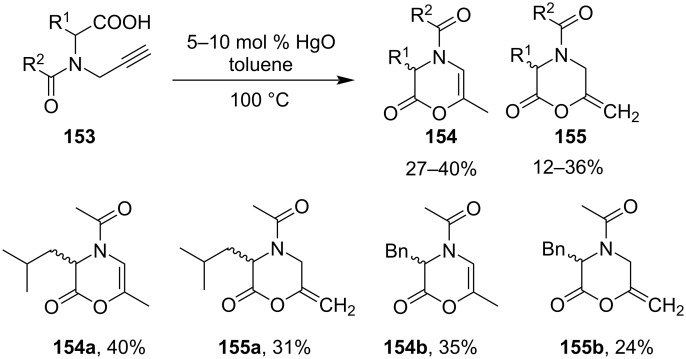
Six-membered morpholine derivative formation by catalytic Hg(II)-salt-induced cyclization.

Six-membered morpholine derivatives were also synthesized by catalytic Hg(II)-salt-induced cyclization. Yamamoto and co-workers published the intermolecular cyclization of alkynyl-carboxylic acid **153** to produce 6-membered morpholine type ring compound **154** and compound **155** [103]. The stereochemistry of the chiral amino acid was not conserved in the cyclized product hence it leads to the formation of racemic products with moderate yields (Scheme 44).

**Scheme 45:**
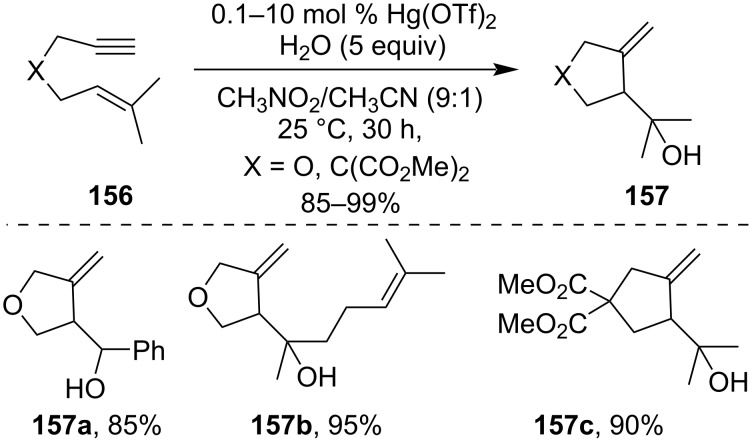
Hg(OTf)_2_-catalyzed hydroxylative carbocyclization of 1,6-enyne.

1,6-Enynes **156** underwent smooth hydroxylative carbocyclization in the presence of catalytic Hg(OTf)_2_. As an example, 3-methylene five-membered cyclic derivatives **157** were synthesized by Nishizawa et al. via the Hg(OTf)_2_-catalyzed hydroxylative carbocyclization of 1,6-enyne **156** (Scheme 45) [104]. It was observed that from 1,7-enynes, six-membered rings were formed in low to moderate yield while from 1,8-enynes, uncyclized hydrated products were isolated. as major products along with different byproducts.

**Scheme 46:**
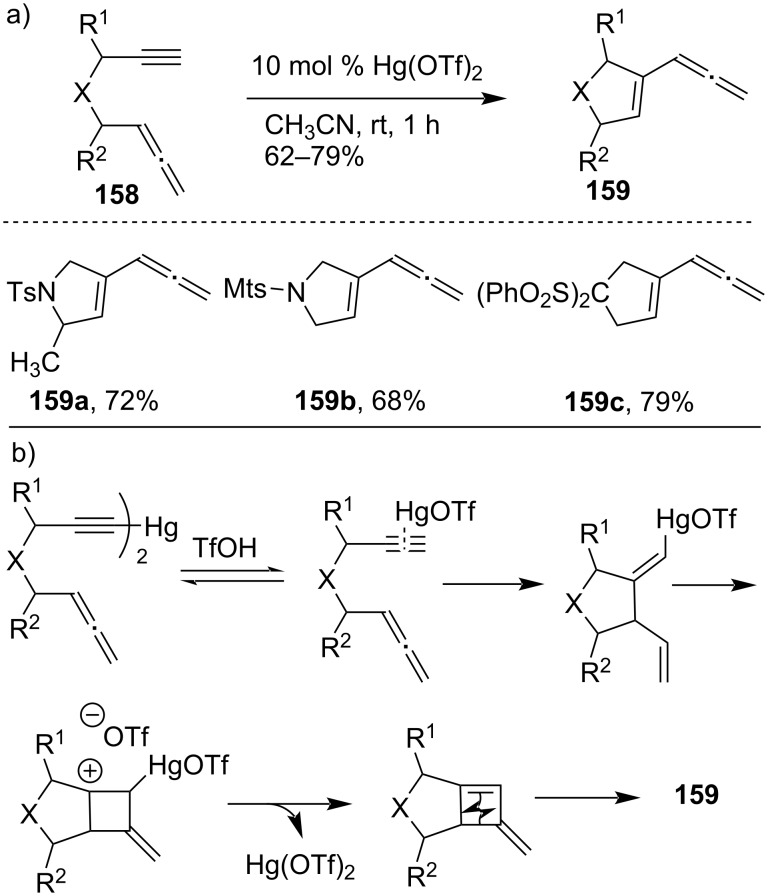
a) Hg(OTf)_2_-catalyzed hydroxylative carbocyclization of 1,6-enyne. b) Proposed mechanism.

In a Hg(OTf)_2_-catalyzed process, 1,6-allenynes **158** were cycloisomerized to generate allenenes **159** in moderate to good yield (Scheme 46) [105]. However, depending on the substituents, allenene and/or unexpected triene were produced as a main product for disubstituted 1,6-allenynes. It was hypothesized based on experimental evidence that alkynes would first form a π-complex with Hg(OTf)_2_, followed by vinylmercuration, demercuration, and eventually isomerizes to allenene.

**Scheme 47:**
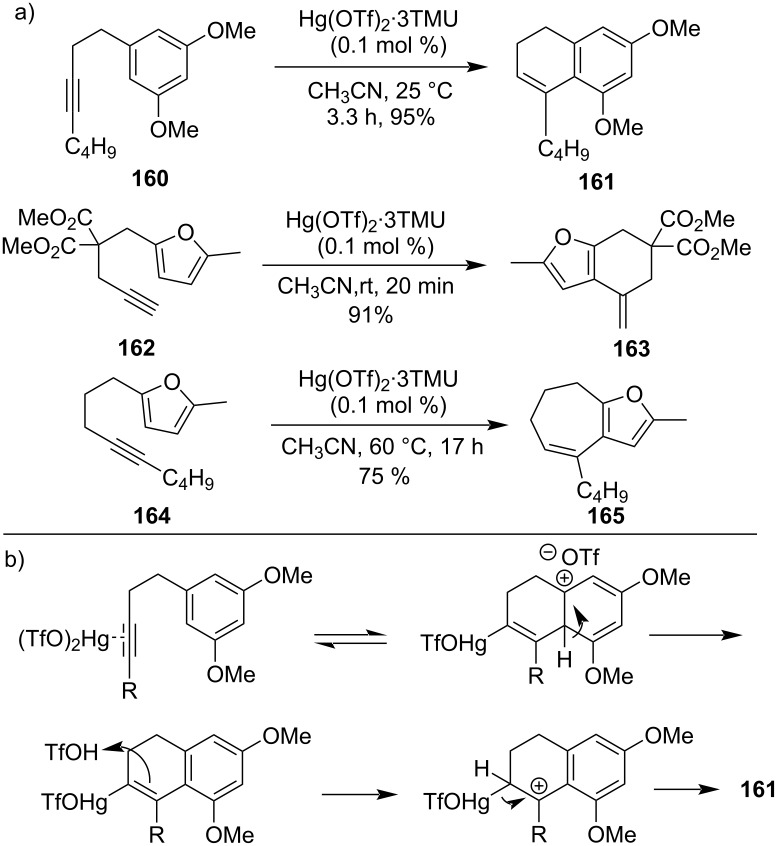
a) Synthesis of carbocyclic derivatives using a catalytic amount of Hg(II) salt. b) Proposed mechanism for dihydronapthanene formation.

In 2003, a carbocyclization with a catalytic quantity of mercury salt was used to efficiently synthesize dihydronapthalene derivative **161** from exemplifying benzyl derivative **160** [106]. The reported methodology was an example of the Hg(OTf)_2_·(TMU)_3_ complex promoting a moderate and efficient procedure for arylalkyne cyclization to directly afford dihydronapthalene derivatives (Scheme 47). Later, Friedel–Crafts type reaction of alkynylfuran **162** and **164** were reported in presence of the Hg(OTf)_2_·0.1 Sc(OTf)_3_ complex (5 mol %) to form six- (**163**) and seven-membered rings (**165**) in good yield. For the cyclization at 2-position of the furan the Hg(OTf)_2_·(TMU)_3_ complex was used as catalyst [107].

**Scheme 48:**
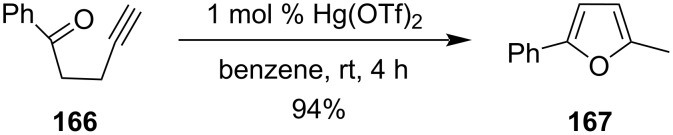
Cyclization of 1-alkyn-5-ones to 2-methylfuran derivatives.

Later it was reported that 1-alkyn-5-ones such as **166** also undergo an effective cyclization reaction to synthesize 2-methylfuran derivatives **167** with high yield in the presence of Hg(OTf)_2_ as the catalyst under very mild reaction conditions (Scheme 48) [108].

**Scheme 49:**
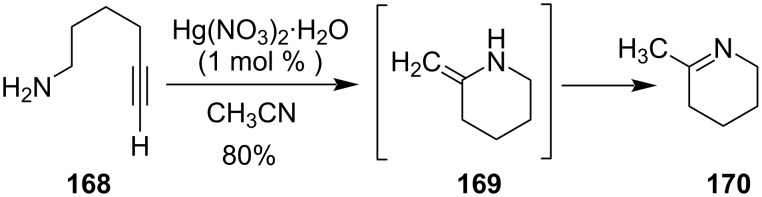
Hg(NO_3_)_2_-catalyzed synthesis of 2-methylenepiperidine.

Cyclization of 6-aminohex-1-yne **168** was performed by catalytic amounts of Hg(NO_3_)_2_ and generated 2-methylenepiperidine **169** initially, which further isomerizes to form 2-methyl-1,2-dehydropiperidine (**170**, Scheme 49) [109].

**Scheme 50:**
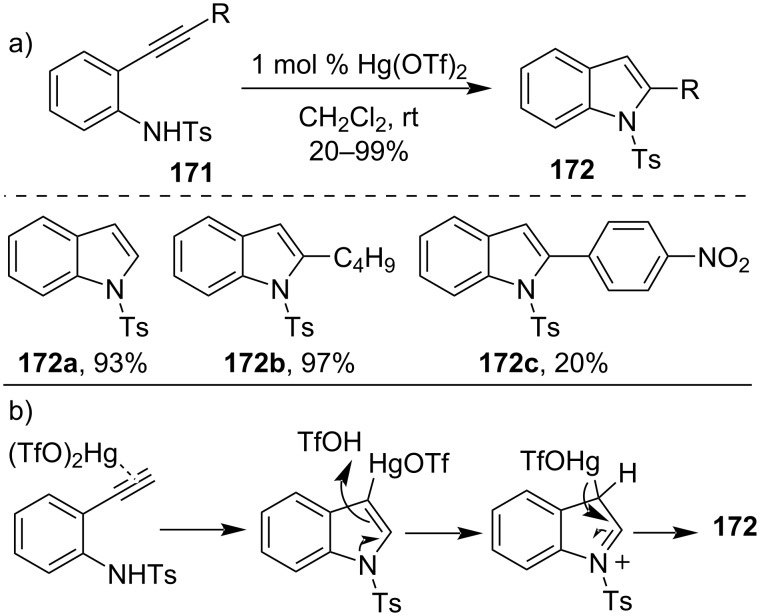
a) Preparation of indole derivatives through cycloisomerization of 2-ethynylaniline and b) its mechanism.

Similarly, in 2007 Kurisaki et al. had also prepared indole derivatives **172** with excellent yields from 2-ethynylaniline derivatives **171** upon the treatment of catalytic amounts of Hg(OTf)_2_ at room temperature. It was an example of cycloisomerization of 2-ethynylaniline derivatives utilizing mild reaction conditions (Scheme 50) [110].

**Scheme 51:**
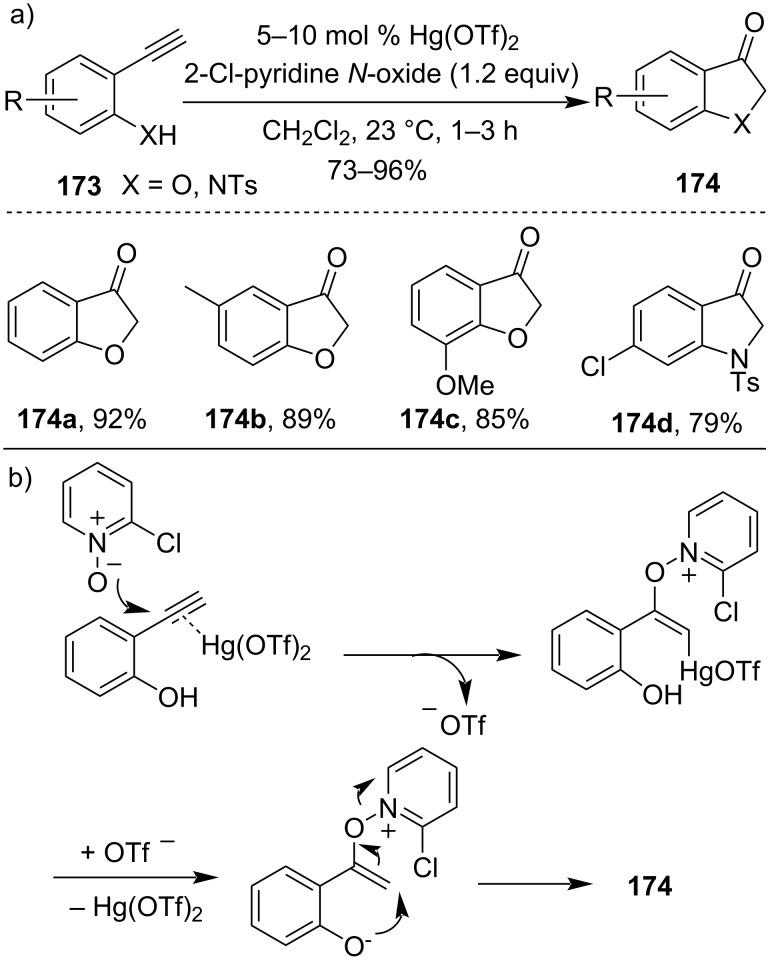
a) Hg(OTf)_2_-catalyzed synthesis of 3-indolinones and 3-coumaranones and b) simplified mechanism.

Rong et al. had demonstrated the Hg(II)-salt-catalyzed enolate umpolung reaction for the efficient synthesis of various 3-indolinones and 3-coumaranones **174**. They had proved that the reaction mechanism proceeds via activation of the alkynyl group with Hg(OTf)_2_ salt and addition of 2-chloropyridine *N*-oxide. The resulting activated alkynyl complex was demercurated, followed by the SN_2_′ reaction thus formed undergoes demercuration to yield 3-coumaranone (Scheme 51) [111].

**Scheme 52:**
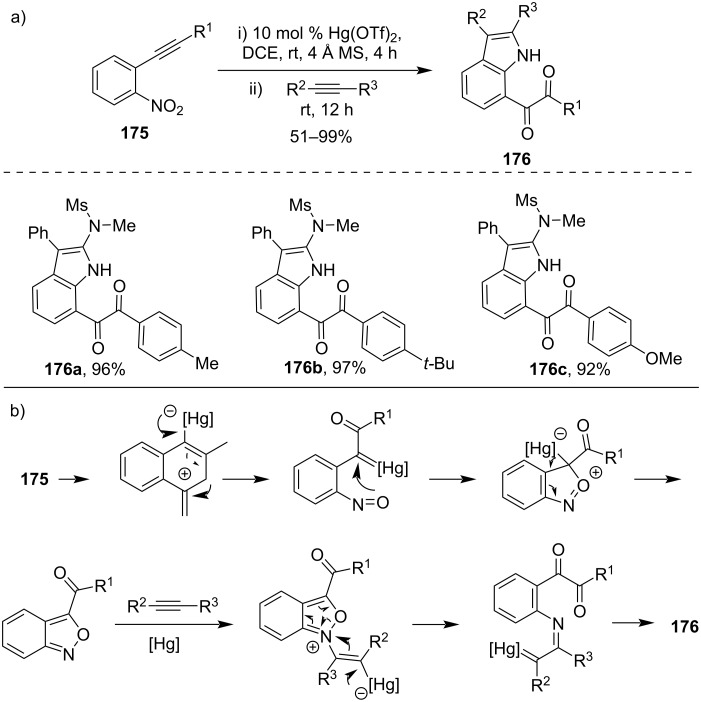
a) Hg(OTf)_2_-catalyzed one pot cyclization of nitroalkyne and b) its plausible mechanism.

Recently, a Hg(OTf)_2_-catalyzed one-pot cyclization of nitroalkyne **175** and alkyne had been reported to synthesize indole derivatives **176**. Based on this strategy, the one-pot method to synthesize indole derivatives had been developed [112]. Similarly, benzo[*c*]isoxazole was also formed in excellent yields with high selectivity using this strategy. In these transformations, two Hg-carbene intermediates were proposed to be involved (Scheme 52).

**Scheme 53:**
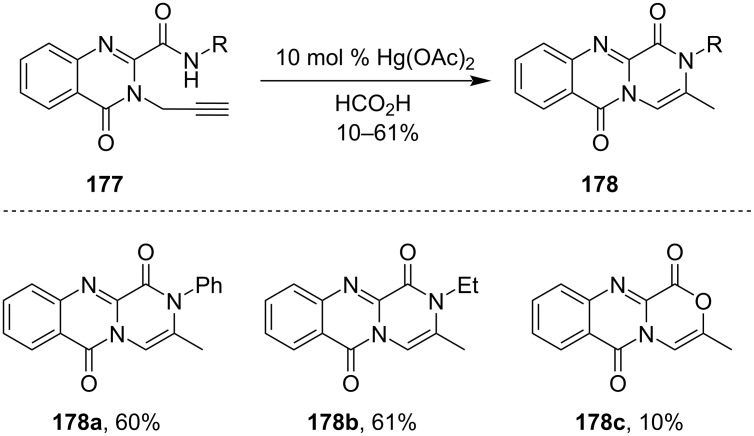
Synthesis of tricyclic heterocyclic scaffolds.

Mercury-catalyzed reactions were also well known for the formation of various complex scaffolds like tricyclic pyrazinones from the corresponding starting materials. For example, Zhang et al. showed that refluxing anilide **177** in presence of a catalytic amount of Hg(OAc)_2_ and 90% formic acid gave the tricyclic heterocyclic scaffold **178** [113]. It involved a two-step process with the rearrangement of the primary cyclization products (Scheme 53).

**Scheme 54:**
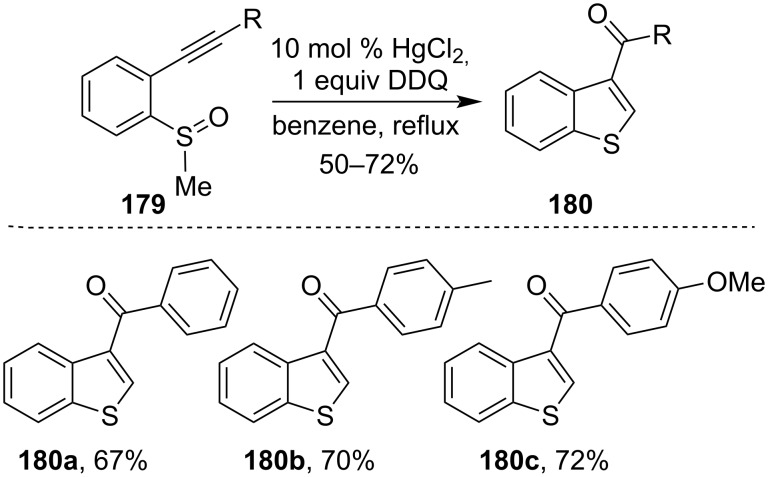
HgCl_2_-mediated cyclization of 2-alkynylphenyl alkyl sulfoxide.

In 2013, Lin et al. reported a Hg(II) chloride-mediated cyclization reaction of 2-alkynylphenyl alkyl sulfoxide **179** to synthesize benzothiophene derivatives **180** with good yields [114]. In this case, the reaction was believed to proceed via the initial formation of metal carbenoids followed by a sequential C–H insertion and then oxidation (Scheme 54). This methodology was later successfully utilized for the total synthesis of raloxifene and benzo[*b*]thiophene derivatives [115].

#### Cyclization involving allenes (>C=C=C<)

**Scheme 55:**
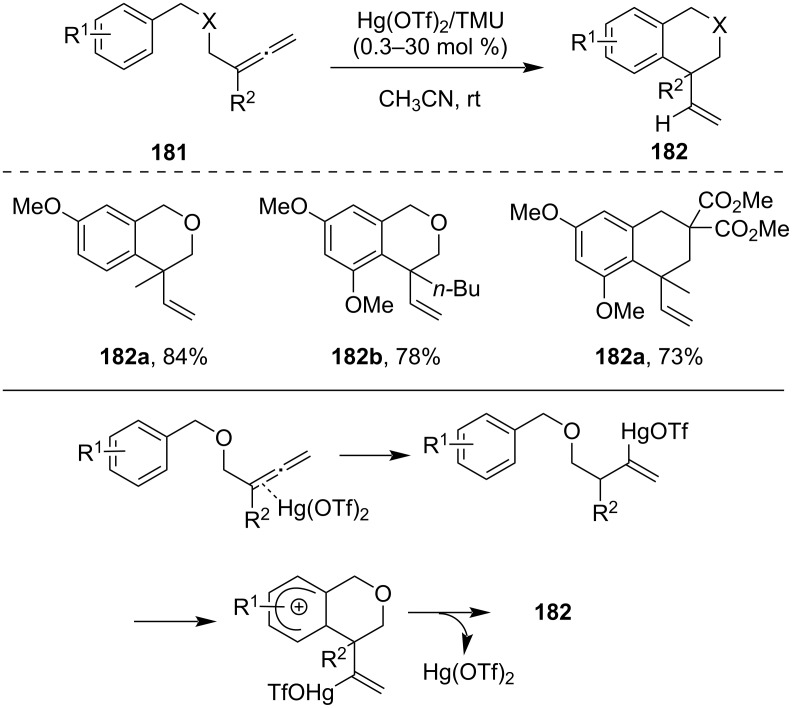
a) Hg(OTf)_2_-catalyzed cyclization of allenes and alkynes. b) Proposed mechanism of cyclization.

Hg(II) triflate salts had also been successfully employed for the arylallene **181** cyclization by Yamamoto et al [116]. The catalytic pathway was proved to involve the direct H-transfer to the vinylmercury complex from the aromatic ring. It involved Hg(OTf)_2_-catalyzed cyclization of aryl 1,1-disubstituted allenes with the formation of a quaternary carbon center followed by the formation of a cationic vinylmercury intermediate (Scheme 55).

**Scheme 56:**
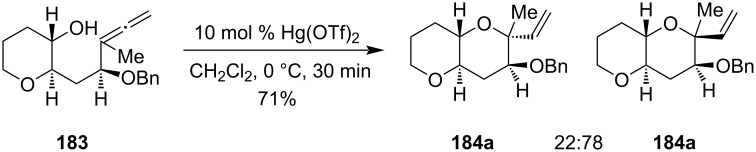
Stereoselective synthesis of tetrahydropyran derivatives.

For the synthesis of stereoselective tetrahydropyran derivatives **184**, Hg(II)-catalyzed cyclization proved to be more effective than silver(I)-salt-mediated cyclization. It showed that methyl-substituted allenes undergo efficient cyclization to form polycyclic ethers under Hg(OTf)_2_-catalyzed conditions at lower temperature (Scheme 56) [117].

**Scheme 57:**
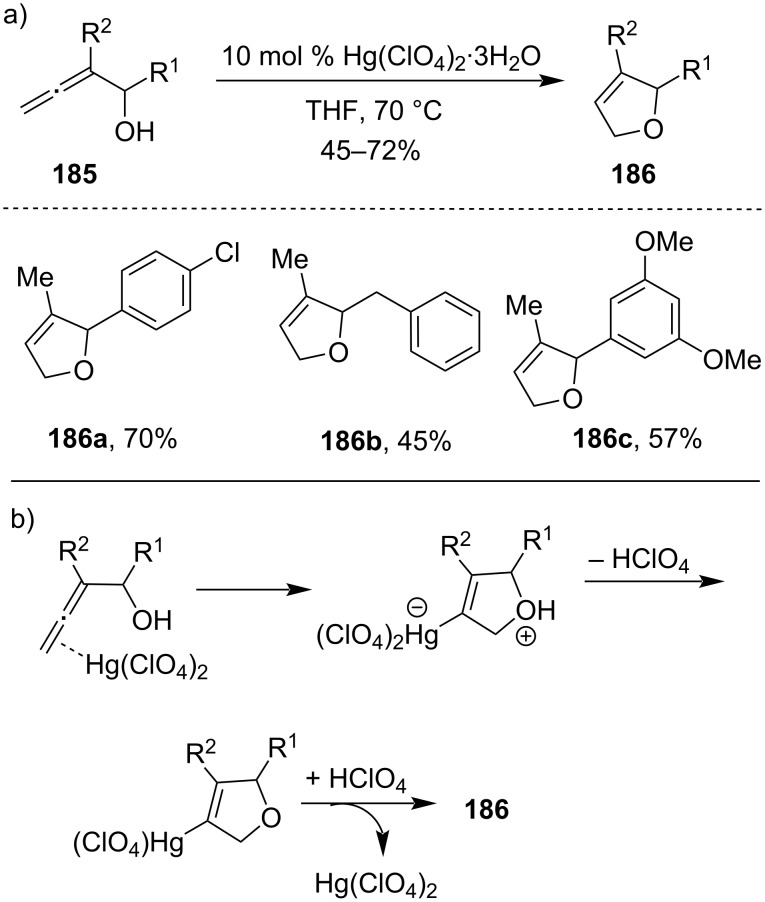
a) Hg(ClO_4_)_2_-catalyzed cyclization of α-allenol derivatives. b) Simplified mechanism.

Following a similar strategy, mercury chlorate (Hg(ClO_4_)_2_) had been employed successfully as a cheap alternative to precious metals salts in the cyclization of α-allenol derivatives **185** to differently substituted 2,5-dihydrofurans **186** in an efficient and selective manner. It was also shown that from enantiopure allenyl derivatives, the desired pure cyclized product was generated by utilizing the above reaction conditions (Scheme 57) [118].

### Mercury(II)-salt-mediated cyclization in total synthesis

Apart from previously described cyclization reactions, there were examples where a Hg(II)-salt-mediated cyclization had been successfully employed as one of the important steps during the total synthesis of natural products.

**Scheme 58:**
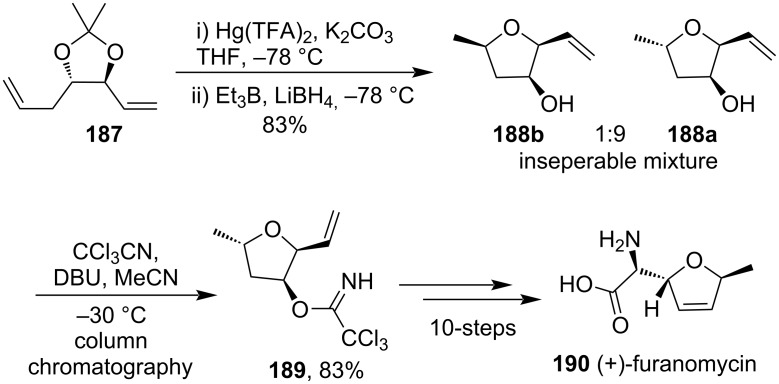
Hg(TFA)_2_-promoted cyclization of a γ-hydroxy alkene derivative.

In 1998, a highly enantioselective total synthesis of (+)-furanomycin (**190**) was achieved through Hg(TFA)_2_-promoted cyclization of γ-hydroxyalkene derivative **187** as an intermediate stage to give a mixture of diastereomeric 2,5-disubstituted tetrahydrofurans **188a** and **188b** (Scheme 58) [119].

**Scheme 59:**
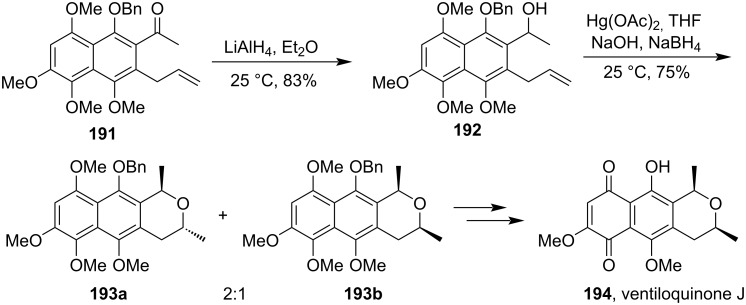
Synthesis Hg(II)-salt-mediated cyclization of allyl alcohol for the construction of ventiloquinone J.

Later for the total synthesis of ventiloquinone J (**194**), a Hg(II)-salt-catalyzed intramolecular cyclization reaction of the *ortho*-allyl alcohol **192** was involved. The reaction went through the formation of a mixture of diastereoisomers **193a** and **193b**. After which the inseparable mixture of products underwent further oxidative demethylation and yielded the final products (Scheme 59) [120].

**Scheme 60:**
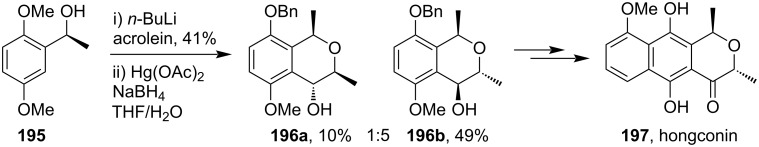
Hg(OAc)_2_-mediated cyclization as a key step for the synthesis of hongconin.

Kraus and co-workers had reported the synthesis of the racemic naphthohydroquinone hongconin (**197**) starting from the *ortho*-allyl alcohol derivative **195**. The starting material was cyclized using a Hg(II) salts to get an inseparable mixture of products **196a** and **196b** in the ratio of 1:5 [121]. The synthetic route proceeded by benzylic alcohol *ortho*-metalation followed by a regioselective mercuri-cyclization reaction (Scheme 60).

**Scheme 61:**
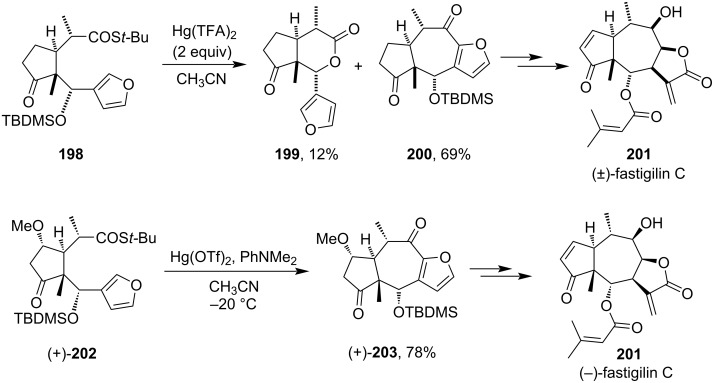
Examples of Hg(II)-salt-mediated cyclized ring formation in the syntheses of (±)-fastigilin C and (−)-fastigilin C.

During the total syntheses of (±)-fastigilin C and (–)-fastigilin C (**201**), 2 equiv of Hg(TFA)_2_ were used to synthesize key intermediate tricyclic furan compounds **199**, **200**, and **203**. Hg(TFA)_2_ helped in the desired ring-formation reaction of compound **198** to afford two cyclic compounds **199** and **200** in the ratio of 6:1 with an overall 81% yield [122]. But in the case of methoxy-substituted derivative **202** only one furan derivative **203** was formed (Scheme 61).

**Scheme 62:**
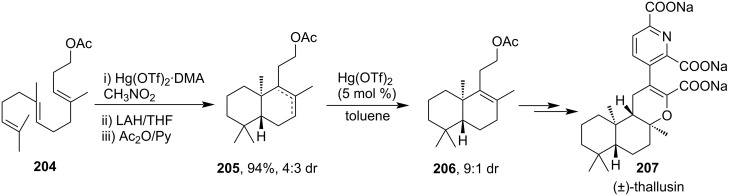
Formal synthesis of (±)-thallusin.

In 2007, Nishizawa et al. successfully utilized a Hg(OTf)_2_-induced cyclization in the key step for the synthesis of (±)-thallusin (**207**, Scheme 62). A complex mixture of Hg(OTf)_2_ and *N*,*N*-dimethylaniline (DMA) (1.2 equiv) was initially used for olefin cyclization to produce a regio- and a stereoisomeric mixture of acetate **205** after reduction and acetylation of the crude product. The Hg(OTf)_2_-catalyzed isomerization of the double bond in compound **205** yielded thermodynamically favorable isomer **206** as a major product [123].

**Scheme 63:**
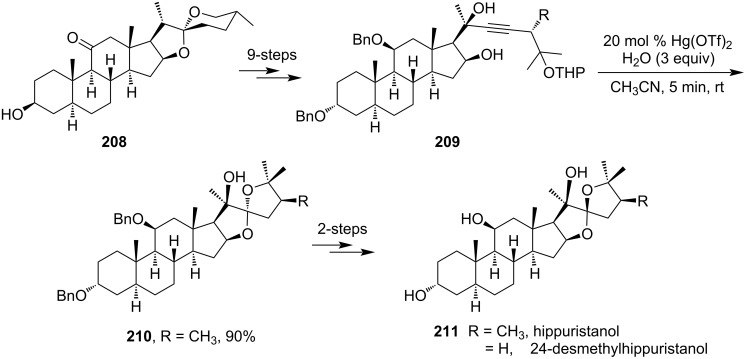
Total synthesis of hippuristanol and its analog.

In 2010, Ravindar et al*.* developed the total synthesis of the steroidal natural product hippuristanol (**211**) starting from 11-ketotigogenin **208** (Scheme 63). They had utilized a Hg(OTf)_2_-catalyzed spiroketalization reaction in their key step to form the desired ketal intermediate **210** [124]. Subsequently, in 2011, the same group reported another synthesis of hippuristanol (**211**) and its analog from easily available starting materials [125]. In this work, hippuristanol and some analogs were successfully synthesized utilizing a Hg(OTf)_2_-catalyzed cascade spiroketalization step of the 3-alkyne-1,7-diol motif. The Hg(OTf)_2_-catalyzed cascade spiroketalization step was proved to be more convenient than Suárez cyclization.

**Scheme 64:**
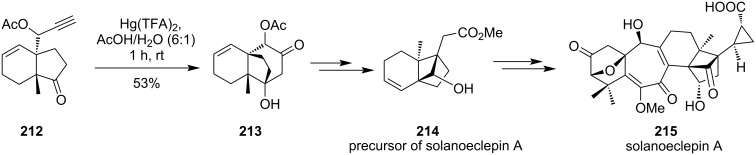
Total synthesis of solanoeclepin A.

A Hg(TFA)_2_-mediated cyclization was efficiently utilized for the synthesis of highly strained tricyclo[5.2.1.0^1,6^]decene intermediate **214** containing a cyclobutane ring (Scheme 64). Compound **213** is an important precursor for the asymmetric total synthesis of solanoeclepin A. The formation of β-hydroxyketone **213** was achieved by Hg(TFA)_2_-mediated cyclization of compound **212** using TFA/H_2_O (1.7: 1) as the solvent. In presence of other mercury compounds like HgO and Hg(OAc)_2_ no product or starting material was recovered [126].

**Scheme 65:**
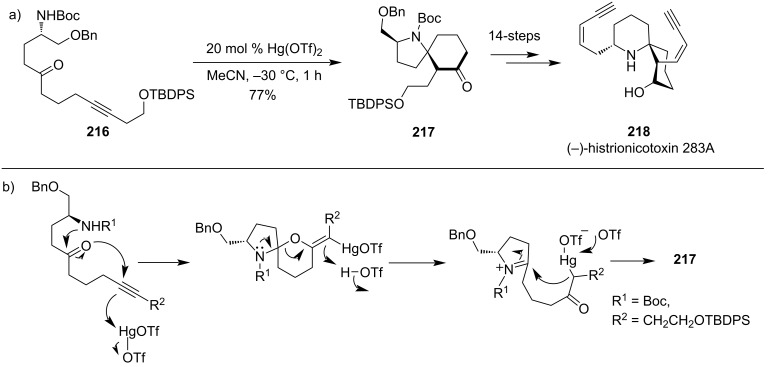
a) Synthesis of Hg(OTf)_2_-catalyzed azaspiro structure for the formation of natural products. b) Proposed mechanism for azaspiro formation.

Spiro-skeleton structures are found in many natural products and synthesizing stereoselective spiro-skeletons has always been difficult for organic chemists. Morimoto and co-workers were the first to disclose the Hg(OTf)_2_-catalyzed cycloisomerization of amino ynone to produce the azaspiro skeleton. Later, this methodology was successfully used for the synthesis of several spiroskeleton structures. Natural products such as histrionicotoxin alkaloids **218** (Scheme 65) [127–128] and lepadoformine [129–130] were being successfully synthesized using this methodology for spirocyclic ring structure synthesis. The proposed mechanism proceeded initially with aminoketal formation by 6-*exo*-dig intramolecular oxymercuration, followed by Petasis–Ferrier-type cyclization, and finally nucleophilic addition of mercuric enolate to iminium results in the formation of azaspiro structure.

## Conclusion

In conclusion, this review summarizes Hg(II)-salt-mediated cyclization reactions either for direct synthesis of cyclized products or as a part of the total synthesis of important natural products. Different Hg(II) salts were used stoichiometrically or catalytically depending upon the nature of functional groups and reactants. However, the reactivity of different unsaturated bonds involved in the cyclization primarily depends on the nucleophile as well as nature of functional groups attached to unsaturated bonds. When alkenes are linked to activating groups like methoxy or hydroxy, a catalytic quantity of Hg(II) ions is required for cyclization. Nothing can be predicted in case of alkynes; however, the presence of a strong nucleophile promotes the Hg(II)-salt-catalyzed cyclization of allenes in most circumstances. In cyclization reactions, Hg(OTf)_2_ showed to be the most effective and versatile of all Hg(II) salts. Mercury(II) salts can also be used to cyclize unsaturated bonds in a regio- and diastereoselective manner. Apart from toxicity concerns, Hg(II) salts are cheap, stable, and versatile in terms of reactivity, making them a viable option to similar transition metal catalysts.
